# A review of the predictors of antimicrobial use and resistance in European food animal production

**DOI:** 10.3389/frabi.2023.1209552

**Published:** 2023-08-04

**Authors:** Carys J. Redman-White, Dominic Moran, Andrew R. Peters, Adrian Muwonge

**Affiliations:** ^1^ Global Academy of Agriculture and Food Systems (GAAFS), The Royal (Dick) School of Veterinary Studies, University of Edinburgh, Edinburgh, United Kingdom; ^2^ Supporting Evidence-Based Interventions in Livestock (SEBI-L), The Royal (Dick) School of Veterinary Studies, University of Edinburgh, Edinburgh, United Kingdom; ^3^ Digital One Health Lab, Roslin Institute, The Royal (Dick) School of Veterinary Studies, University of Edinburgh, Edinburgh, United Kingdom

**Keywords:** antimicrobial resistance (AMR), antibiotic resistance (ABR), veterinary drugs, livestock (including poultry), agriculture, one health (OH), animal husbandry and aquaculture, sustainable food and agriculture policy

## Abstract

Antimicrobial resistance (AMR) is a major threat to global health and a key One Health challenge linking humans, animals, and the environment. Livestock are a key target for moderation of antimicrobial use (AMU), which is a major driver of AMR in these species. While some studies have assessed AMU and AMR in individual production systems, the evidence regarding predictors of AMU and AMR in livestock is fragmented, with significant research gaps in identifying the predictors of AMU and AMR common across farming systems. This review summarizes existing knowledge to identify key practices and critical control points determining on-farm AMU/AMR determinants for pigs, layer and broiler hens, beef and dairy cattle, sheep, turkeys, and farmed salmon in Europe. The quality and quantity of evidence differed between livestock types, with sheep, beef cattle, laying hens, turkeys and salmon underrepresented. Interventions to mitigate both AMU and/or AMR highlighted in these studies included biosecurity and herd health plans. Organic production typically showed significantly lower AMU across species, but even in antibiotic-free systems, varying AMR levels were identified in livestock microflora. Although vaccination is frequently implemented as part of herd health plans, its effects on AMU/AMR remain unclear at farm level. Social and behavioral factors were identified as important influences on AMU. The study fills a conspicuous gap in the existing AMR and One Health literatures examining links between farm management practices and AMU and AMR in European livestock production.

## 1 Introduction

AMR is a “silent pandemic” and a quintessential One Health challenge spanning human, animal and environmental health (Robinson et al., 2016). Our understanding of the contribution of human and veterinary AMU and environmental contamination with antimicrobials to overall burden is imperfect, and there is a need to understand the drivers as part of the epidemiology of AMR. While the relationship between AMU in livestock and AMR in humans is not fully understood, the impact of AMR on human health suggests that AMR in livestock could have similar negative impacts on animal health, which has intrinsic as well as economic value. Recent estimates suggest that, by weight, most of the world’s antibiotics are used in livestock production and the consumption of antimicrobials on farms is predicted to grow rapidly in line with rising livestock populations (Van Boeckel et al., 2017; Tiseo et al., 2020). However, the literature on specific factors affecting both AMU and AMR in livestock is fragmented, with no overall summary of the entry points for changing practice in different animal production systems to minimize the requirement for AMU, avoid unnecessary AMU, and mitigate AMR. This paper addresses this gap by drawing together published evidence in a review of the critical control points that determine the factors affecting AMU within farming systems and evidence of that use as a driver of AMR. The review covers pigs, sheep, dairy and beef cattle, broiler and layer chickens, turkeys and salmon. It predominantly covers European systems, although a discussion offers comparisons with other high-income countries and observations on the likely relevance to lower-income country smallholder production.

A variety of observational studies of putative drivers of AMU and AMR on farms have been carried out, typically covering one or sometimes two species and generally one country. Other, multi-partner collaborations have facilitated pan-European epidemiological and metagenomic studies (EFFORT Consortium, 2014). Several intervention studies have also investigated the effects of specific factors such as dietary content or stocking density on AMU and/or AMR. Qualitative and quantitative socioeconomic studies have identified attitudes and beliefs affecting AMU among livestock veterinarians and farmers (Golding et al., 2019; Skjolstrup et al., 2021). AMR studies have typically cultured indicator species such as *E. coli* to measure AMR, but with increasing availability of next-generation sequencing, metagenomic studies are also being used to investigate resistomes.

Previous reviews have addressed some aspects of farm management and their impacts on AMU or AMR, but with limited scope. Some focused on management factors influencing overall health in a production system (Bessei, 2006); others looked at either a subset of AMR risk factors (Davies and Wales, 2019), or risk factors for presence of specific AMR organisms (Becker et al., 2021). One review addressed AMU risk factors in veal calves, pigs and poultry but did not include AMR (Bokma et al., 2018). Growing interest in AMU and AMR in livestock has led to a considerable increase in research evidence published subsequent to that review, published in 2018. Larger scale analyses have modelled AMU and AMR at a global scale in livestock (Van Boeckel et al., 2015; Van Boeckel et al., 2019; Tiseo et al., 2020) and one study examined socioeconomic correlates of AMR in livestock and humans at the country level (Allel et al., 2023).

This review is designed to answer the research question, “What farm-level practices and characteristics are associated with increased AMU and AMR in European food animal production?” It is structured to address the distribution of literature among livestock types, before summarizing evidence for the impacts of different management practices and other potential influences on AMU and AMR for each livestock type, in order to identify specific research gaps and opportunities for intervention.

## 2 Methods

Search terms were assembled according to four themes: species of interest, AMU and AMR terms, terms relating to production types and husbandry and terms relating to the location of interest. Terms within each theme were combined using the “OR” operator and the themes were combined using the “AND” operator (**Table 1**). A keyword search was carried out in the Web of Science and Scopus databases and an equivalent search was carried out using Medical Subject Headings (MeSH) in the MEDLINE database. To meet acceptance criteria, papers had to be observational or intervention studies relating to beef cattle, dairy cattle, pork, laying chickens, broiler chickens, broiler turkeys, sheep, or farmed salmon in European production systems. AMU or AMR had to be included as an outcome variable, with production system, management, farmer or veterinarian characteristics, or husbandry attributes as independent variables. Only English-language publications were included. Whilst interest in AMU and AMR in livestock has grown over the years, reflected in an increase in relevant studies in recent years, all publication dates up until the search itself was carried out in May 2022 were included, facilitating qualitative comparison of studies carried out before and after major livestock policy changes such as cessation of antimicrobial growth promotion.

**Table 1:** Keywords and MESH headings used in searches.

	Species of interest	AMU/AMR terms	Production types and husbandry	Location
**Keywords**	Livestock Pigs Porcine Poultry Poults Broiler Turkeys “Laying hen” Layer Chicken Flock Ruminant Cattle Dairy Beef Bovine Sheep Lamb Ovine Aquaculture Salmonid Salmon	antimicrobial “antimicrobial resistant” “antimicrobial resistance” AMR AMU antibiotic “antibiotic resistant” “antibiotic resistance” “drug resistant” “drug resistance”	“Production system” “Farm management” Intensive Extensive Organic Conventional Farrowing Calving Lambing Spawn Biosecurity Grower Weaner Finisher Breeding Grazing “Free range” Barn Caged Stalls “Stocking density” Housing “Sea pens” “Freshwater pens” “Mixed farm” Smallholding “Backyard chickens” “Backyard poultry”	“European Union” EU Europe Britain “United Kingdom”
**MESH headings**	livestock/ poultry/ chickens/ turkeys/ salmo salar/ cattle/ sheep/ sus scrofa/	drug resistance, bacterial/ anti-bacterial agents/	animal husbandry/ fisheries/ dairying/ farms/ organic agriculture/	Europe/ european alpine region/ andorra/ austria/ balkan peninsula/ belgium/ europe, eastern/ france/ germany/ gibraltar/ united kingdom/ greece/ ireland/ italy/ liechtenstein/ luxembourg/ mediterranean region/ monaco/ netherlands/ portugal/ san marino/ “scandinavian and nordic countries”/ spain/ switzerland/ transcaucasia/

**Figure 1:**
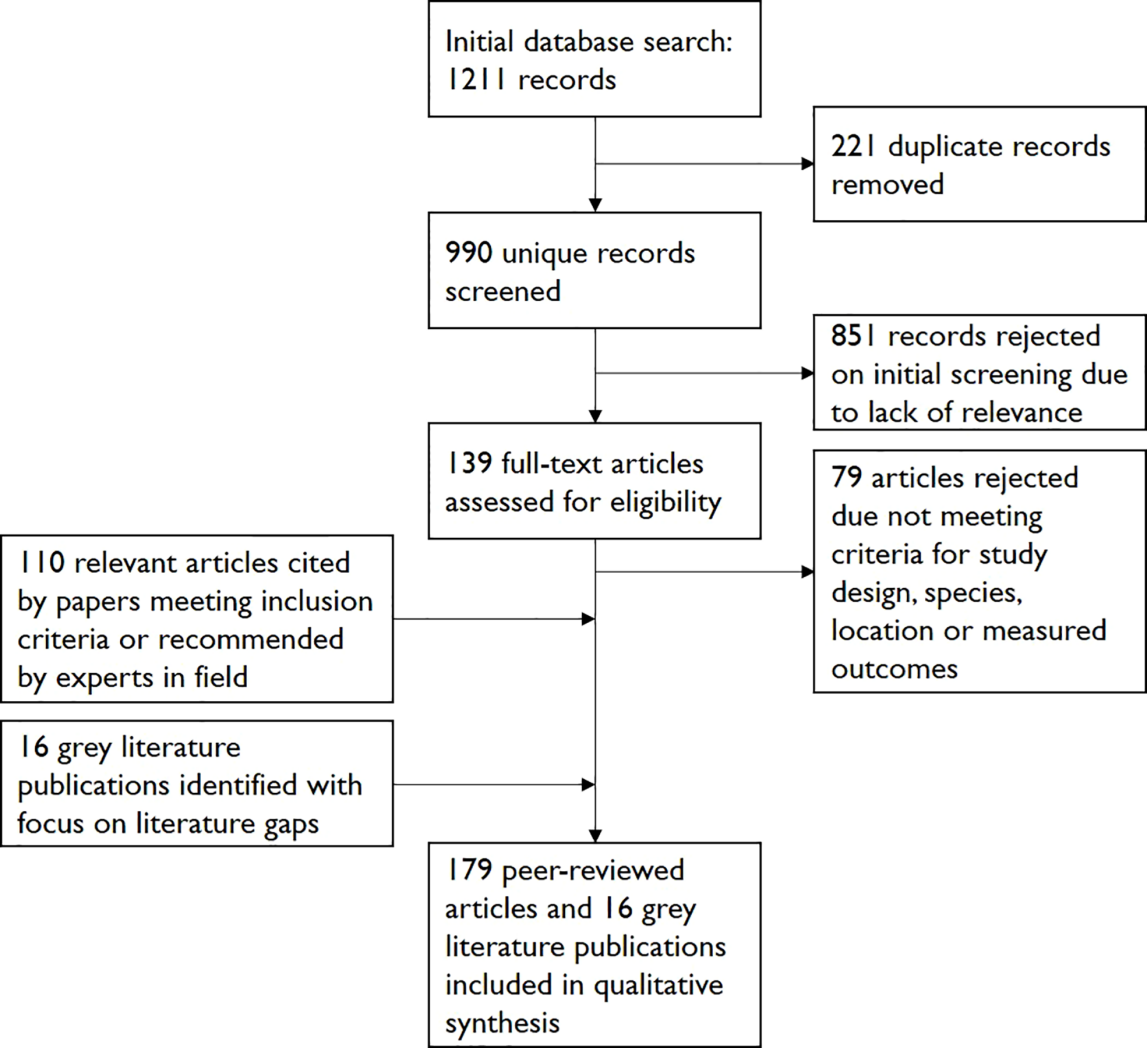
Literature search and origin of material included in review.

These searches yielded 1211 papers in total. 221 duplicates were removed and 851 papers were rejected on initial screening. Of the remaining 139 papers, 79 were rejected on further reading due to falling outside the above criteria. Discussion with experts in the field, particularly focusing on literature gaps, guided grey literature searches and helped identify further relevant peer-reviewed studies and grey literature. The reference lists of the accepted papers were assessed to identify further relevant papers and grey literature publications as part of a “snowballing” strategy. A total of 179 peer-reviewed studies and 16 grey literature publications were included in the final review (see **Figure 1**).

The content of the material included in the review was summarized in a spreadsheet (**Supplementary Material**), including location and year of data collection, livestock types, study aim, research design, type of data collected, sample sizes, independent variables, and metrics used for AMU, AMR and other outcomes. Key findings, with p-values, were summarized for each study, including potential predictors (defined here as farm-level factors statistically associated with each) of AMU and AMR, noting where evidence of associations with AMU or AMR was weak or conflicting as assessed by the authors. Strength of evidence was judged based on study design, concordance of findings (within and between studies), and statistical significance of results. These predictors were categorized into internal and external biosecurity, feed and housing, farmer decision-making (e.g., whether managing the farm as an organic system), livestock traits, and factors specific to the livestock type (e.g. milking practices for dairy cattle). Each category of predictor was mapped onto a diagram of the production cycle incorporating relevant inputs and outputs and the points in the production cycles identified by the publications as peak AMU points were annotated.

## 3 Results

### 3.1 Publications

**Figure 2:**
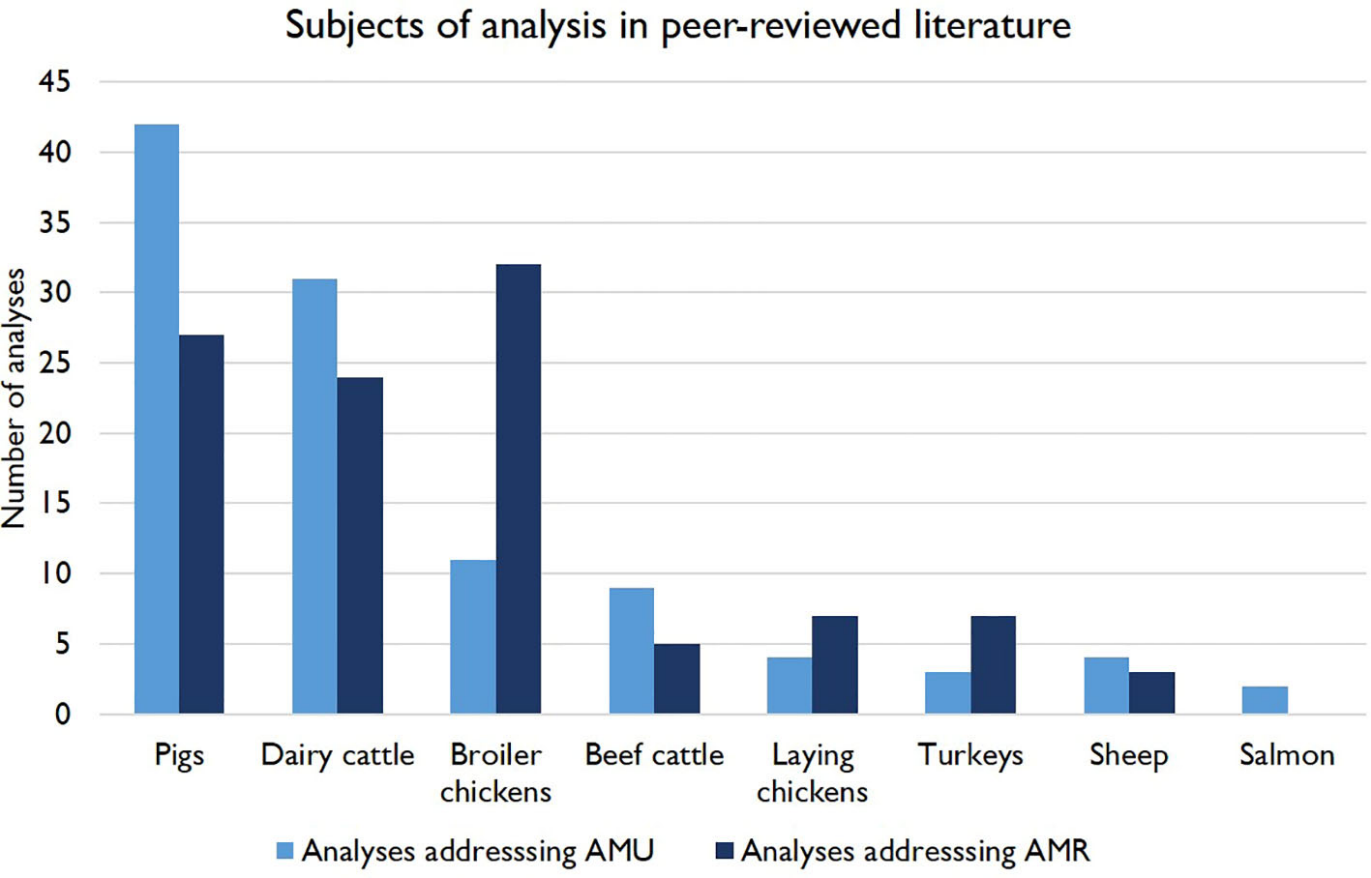
Number of peer-reviewed studies included in this review addressing predictors of AMU and AMR in different categories of farmed livestock in Europe. Some publications are represented more than once, as they had both AMU and AMR as outcomes and/or addressed multiple livestock types.

Pigs, dairy cattle and broiler chicken production are most strongly represented in the literature in this area, with 66, 52 and 43 peer-reviewed papers addressing these livestock categories, respectively. Only 14 papers were identified addressing beef cattle, 10 for laying chickens, 7 for sheep, 9 for broiler turkeys and 2 for farmed salmon (**Figure 2**). 24 publications covered multiple livestock types and the results of one survey of organic farmers were aggregated across species (Chylinski et al., 2022). The relative impacts of organic systems on AMU and/or AMR were a common topic of investigation (n = 11), and to a lesser extent free-range farming in general. Biosecurity was also addressed in many studies, with the Biocheck.UGent tool (Gelaude et al., 2014) used by many papers (n = 18) as a standardized measurement. Behavioral and social predictors of veterinarians’ and farmers’ AMU practices were addressed in the literature (n = 27), as were herd health planning and preventative treatments (n = 16). The potential impact of management influences on AMR mediated through the gut microbiome was an emerging area of research (n = 6), including investigations of impacts of taxonomic variation on AMR and trials assessing dietary predictors and applications of competitive exclusion (CE) flora to mitigate both AMU and AMR. Some practices investigated were specific to production systems, with a large proportion of the dairy cattle literature focusing on antimicrobial dry cow therapy (DCT) (n = 17) and the feeding of antibiotic-containing waste milk to calves (n = 10).

Details for all reviewed publications are presented in the **supplementary materials**, including country and year(s) of data collection, study design, sample sizes, and key findings including p-values where reported.

### 3.2 Dairy cattle

Available UK datasets give differing estimates of mean farm-level AMU, at 15.5 and 19.7 mg/kg PCU (FarmAssist, 2021; Kingshay Independent Dairy Specialists, 2021) and suggest that, as with other livestock production systems across Europe, the distribution of AMU among dairy farms is right-skewed (Kingshay Independent Dairy Specialists, 2021; Responsible Use of Medicines in Agriculture Alliance (RUMA), 2021). They indicate that a small number of production units account for a disproportionately large volume of AMU, with the individual farms in the top quartile for AMU changing from year to year (Kingshay Independent Dairy Specialists, 2021). This right-skew was not seen in national level dairy cattle data for the Netherlands, although the pattern was noted in other species in the same report (Van Geijlswijk et al., 2019). Further investigation is needed to identify whether right-skew is the norm in other countries and internationally.

#### 3.2.1 Organic status

Organic dairy farms generally demonstrated lower AMU than conventional systems across three European studies (Alliance to Save Our Antibiotics, 2021; Bennedsgaard et al., 2010; Antillon et al., 2020), with data from the UK showing evidence of a right-skewed distribution of AMU in organic farms similar in shape to that seen in available data for the dairy sector nationally (Veterinary Medicines Directorate (VMD), 2020), with mean AMU approximately 1.5 times the median value (Alliance to Save Our Antibiotics, 2021).

Findings typically reflected more selective use of DCT on organic farms (Brunton et al., 2012; Poizat et al., 2017), but these differences were not always significant (Firth et al., 2019; Antillon et al., 2020). A 2010 survey of British dairy farmers reported that organic farmers were less likely to use blanket DCT, antimicrobial treatment of all cows at the end of lactation, although no statistical analysis was reported. The study suggested similar use of highest priority critically important antibiotics (HP-CIAs) by the two groups (Brunton et al., 2012). However, a 2016 UK survey found organic dairy farms less likely than conventional farms to include HP-CIAs in their three most frequently used veterinary medicines (Higham et al., 2018). Where they were measured, health outcomes such as udder health were not negatively affected by lower AMU (Hamilton et al., 2006; Valle et al., 2007).

Impacts of organic farming on AMR are uncertain. No clear differences in AMR were detected between streptococcal and staphylococcal isolates from organic and conventional milk in studies in Switzerland (Busato et al., 2000; Roesch et al., 2006) or Sweden (Sjöström et al., 2020). In a Swiss study, calves from organic farms had significantly higher odds of carrying commensal *E. coli* resistant to several antimicrobials including kanamycin and ampicillin, but extended-spectrum beta-lactamase (ESBL) *E. coli* was found only in calves from conventional farms (Nüesch-Inderbinen et al., 2022).

#### 3.2.2 Farm characteristics

A UK industry analysis of a convenience sample representing approximately 9% of UK herds found weak positive or insignificant correlations between herd size and/or milk yield and AMU (Kingshay Independent Dairy Specialists, 2021). In Finland, blanket DCT was found to be a minority practice significantly associated with larger herds as well as higher milk production (Vilar et al., 2018). Use of automatic milking systems (AMS) was significantly associated with blanket DCT in Finnish farms (Vilar et al., 2018), although there was no significant difference in AMU between AMS and conventional milking systems in a Dutch study (Deng et al., 2020). In a UK study of milk microbiology, use of AMS rather than conventional milking systems was a significant predictor of resistance (McLaughlin et al., 2022). AMR prevalence in fecal or environmental *E. coli* was found to be significantly lower in smaller herds in Sweden (De Verdier et al., 2012) and, in Germany, in farms with traits, traits associated with lower intensity farming, such as longer fattening periods and less farm mechanization (Hille, 2017).

Veterinary treatment frequency for mastitis was significantly higher in Swedish herds with an increased proportion of first-parity cows (Nyman et al., 2007), while cows in a UK study were significantly more likely to be treated with antimicrobials in the first 30 days of lactation if they were third or later parity (Cook, 2020). In a study of a single UK farm, ESBL *E. coli* was significantly more prevalent in feces of lactating cows compared to the herd overall, and around five times more prevalent in multiparous compared to primiparous cows (Watson et al., 2012). A significantly higher prevalence of antibiotic-resistant *E. coli* was found in calves compared to adult dairy cows in this study as well as in others in Sweden, Germany, the Netherlands and the UK (Watson et al., 2012; Duse et al., 2015; Heuvelink et al., 2019; Schubert et al., 2021; Weber et al., 2021). Calves were found to shed resistant *E. coli* at a much higher level than young stock and adults, with possible explanations including differing selection pressures within the calf enteric environment compared to later stages (Heuvelink et al., 2019).

Breed associations with AMU are uncertain. Swedish herds with Swedish red-and-white cattle, a traditional breed associated with less intensive farming, had a significantly lower incidence of mastitis cases treated by a veterinarian (Nyman et al., 2007), while no breed association was seen in a convenience sample of UK herds using a dairy consultancy service (Kingshay Independent Dairy Specialists, 2021).

#### 3.2.3 Calving and calf management

Odds of treatment with antimicrobials in the first 30 days after calving were significantly higher in UK cows calving in summer or winter compared to autumn, while those treated prophylactically with pegbovigrastim (bovine granulocyte colony stimulating factor, G-CSF) had significantly lower odds of requiring treatment (Cook, 2020). Disinfection of the calving area was positively associated with ESBL *E. coli* prevalence in recently calved cows on German dairies (Weber et al., 2021). Possible explanations for positive associations between disinfection and AMR suggested by these and other authors include poor implementation of disinfection protocols, co-selection for resistance, and increased likelihood of disinfectants being introduced on farms with infectious disease problems (Taylor et al., 2009; AbuOun et al., 2020; Luiken et al., 2022).

Risk factors identified for high AMU in young calves in the Netherlands included keeping calves under eight weeks old on slatted floors rather than non-slatted floors and farmer beliefs that young stock do not require any specific management (Holstege et al., 2018). High-AMU farms in this study had significantly higher rates of respiratory disease and higher probability of a history of *Salmonella* on-farm (Holstege et al., 2018). Use of non-antimicrobial prophylactic treatments, such as those for ketosis, in periparturient cows was associated with reduced risk of AMR in calves in Germany, which may be due to the effects of these treatments or could reflect good farm management (Weber et al., 2021).

Feeding of waste milk to calves, found to be a majority practice in a 2010 UK study (Brunton et al., 2012), is no longer recommended following updated guidelines in 2018 (Lloyd, 2018). 46% of UK dairy farmers reported practicing it in 2016 (Higham et al., 2018), comparable to the 48.3% of Swiss dairy farmers in 2020 (Bernier Gosselin et al., 2022). Waste milk is sometimes pasteurized to kill microbes, but this does not solve the problem of antimicrobial residue persistence, and recontamination of pasteurized milk has been demonstrated (Aust et al., 2013). Waste milk feeding of calves was associated with significantly higher prevalence of AMR in multiple studies (Aust et al., 2013; Brunton et al., 2014; Duse et al., 2014; Maynou et al., 2017a; Maynou et al., 2017b; Weber et al., 2021).

#### 3.2.4 Dry cow management

Use of DCT, especially blanket rather than selective treatment, is associated with higher overall AMU (Nyman et al., 2007; Scherpenzeel et al., 2016; Stevens et al., 2016; Firth et al., 2019). Selective use has been identified by farmers as a key approach to reducing their AMU (Higham et al., 2018). In a Dutch study, selective DCT was found to reduce AMU compared to blanket application, but at the cost of higher somatic cell count (SCC) at the following calving for cows dried off without antimicrobials (Scherpenzeel et al., 2014). Non-antimicrobial teat sealants, a suggested alternative, are reportedly used mainly alongside, rather than instead of, antimicrobial DCT (More et al., 2012). Teat sealant use has also been reported in association with selective application of DCT (Scherpenzeel et al., 2016). Positive associations between antimicrobial DCT and AMR prevalence have been demonstrated in Sweden and the UK (Duse et al., 2014; Schubert et al., 2021; McLaughlin et al., 2022), highlighting antimicrobial DCT use as an area of concern.

#### 3.2.5 Biosecurity

##### 3.2.5.1 Internal biosecurity

Poor hygiene of feed storage was identified as a significant risk factor for high antimicrobial treatment incidence for mastitis in Swedish herds, possibly indicative of overall farm hygiene standards (Nyman et al., 2007). Risk factors for AMR in *E. coli* included on-farm slurry storage (Snow et al., 2012), and poor hygiene at milking (also a risk factor for methicillin-resistant *S. aureus*, MRSA, and enterococcal AMR) (Locatelli et al., 2017; Schnitt et al., 2020; Weber et al., 2021; McLaughlin et al., 2022). Poor hygiene for calf care and housing has also been identified as a risk factor for ESBL-*E. coli* (Snow et al., 2012), although disinfection of calving areas and daily washing of calf feeding equipment were positively associated with prevalence of this *E. coli* phenotype in one study (Weber et al., 2021). Calving pens were identified as a reservoir for resistant *E. coli* on a UK dairy farm (Watson et al., 2012). Presence of pigs on the same farm was identified as a risk factor for MRSA carriage in dairies, with persistence in the environment and possible evidence of transmission between species identified on farms in Belgium, Germany, and Italy (Verhegghe et al., 2013; Locatelli et al., 2017; Schnitt et al., 2020).

##### 3.2.5.2 External biosecurity

No research was found investigating impacts of external biosecurity on AMU in dairy cattle. Risk of ESBL *E. coli* presence was significantly positively associated with introducing new stock without quarantine in a UK study (Snow et al., 2012) and, in the Netherlands, with being located within 2km of a pig farm (Santman-Berends et al., 2017). AMR in *Enterococcus* species isolated from bulk milk tanks in UK farms was significantly positively associated with the practice of bringing breeding bulls onto the farm from outside (McLaughlin et al., 2022).

#### 3.2.6 Herd health

Several approaches to mastitis management were identified for reducing AMU. Identifying mild or subclinical cases and in the first instance managing these with supportive care such as massage or NSAIDs in place of antimicrobials was associated with reduced AMU in multiple studies (Hamilton et al., 2006; Nyman et al., 2007; Stevens et al., 2016; Holstege et al., 2018). A targeted lactating cow treatment protocol piloted in dairy farms in Germany demonstrated reduced AMU associated with the intervention without any negative effect on any of the measured clinical parameters (Schmenger et al., 2020). The protocol included measures such as use of NSAIDs as first-line treatments for mild mastitis and on-farm culture to test for presence of Gram-positive causative agents, for which antibiotic treatment is indicated. Some farms in a Danish study were also reported to use “blinding,” drying-off of affected quarters, to control mastitis, although the impacts of this specific measure were not assessed (Bennedsgaard et al., 2010).

Group treatment with antimicrobials, for example metaphylaxis, is not always indicated, and use of oral and footbath antibiotics on UK farms were found to be significantly associated with the farm being in the top quartile for AMU (Hyde et al., 2017). Disease prevention is an important aspect of reducing AMU and AMR, and Swedish herds free of bovine respiratory syncytial virus (BRSV) had a significantly lower proportion of quinolone-resistant *E. coli* than recently infected herds or those with a long-term steady infection rate (Duse et al., 2021).

#### 3.2.7 Human factors

Social norms among farmers and concerns about clinical symptoms persisting or recurring have been associated with antimicrobial course duration as administered by farmers (Swinkels et al., 2015). Dosing rates may also differ from recommended doses on specific product characteristics (SPCs) (Merle et al., 2014), for example due to difficulty accurately estimating body mass of cows (Van Dijk et al., 2015). Veterinary attitudes, beliefs and social pressure from farmers have been shown to be associated with readiness to prescribe antimicrobials, and significant differences in farmer thresholds for seeking veterinary treatment have been observed between nations with similar policy approaches to AMU and AMR (Espetvedt et al., 2013). In Germany a longitudinal observational study of dairy cattle found that the prescribing veterinarian was a significant predictor of farm-level AMU (Hommerich et al., 2019), while a survey of cattle veterinarians in which the majority of respondents practiced in a country in Europe found that the longer a veterinarian had worked in the cattle industry, the less likely they were to be worried about AMR (Llanos-Soto et al., 2021).

Farmer attitudes and personality traits affecting AMU included: beliefs about AMU and AMR (Swinkels et al., 2015; Scherpenzeel et al., 2016), past experiences (Vilar et al., 2018), social norms (Swinkels et al., 2015), opinions of best management practices (Bennedsgaard et al., 2010; Gussmann et al., 2018; Holstege et al., 2018), and individual “treatment threshold” which is defined as the point at which a farmer will call the veterinarian rather than managing a condition themselves (Nyman et al., 2007; Valle et al., 2007; Holstege et al., 2018; Deng et al., 2020). In a study of Norwegian dairy farms, organic production systems were associated with a higher treatment threshold, with organic farmers taking non-pharmaceutical approaches to mild mastitis in the first instance (Valle et al., 2007).

The role of the veterinarian was highlighted in multiple studies, with veterinarian-farmer relationships impacting farm management and disease prevention programs as well as prescribing treatments (Poizat et al., 2017; Speksnijder et al., 2017). More frequent veterinarian contact was associated with greater knowledge of AMR in the UK (Higham et al., 2018) and herd health programs facilitated by veterinarians working in collaboration with farmers were identified as an important tool to help improve herd health and welfare along with decreasing AMU (Stevens et al., 2016; More et al., 2017; Speksnijder et al., 2017; Higham et al., 2018). A trial of farm-specific management changes developed in collaboration with the farmers themselves demonstrated a significantly reduced AMU in intervention farms (Bennedsgaard et al., 2010).

**Figure 3:**
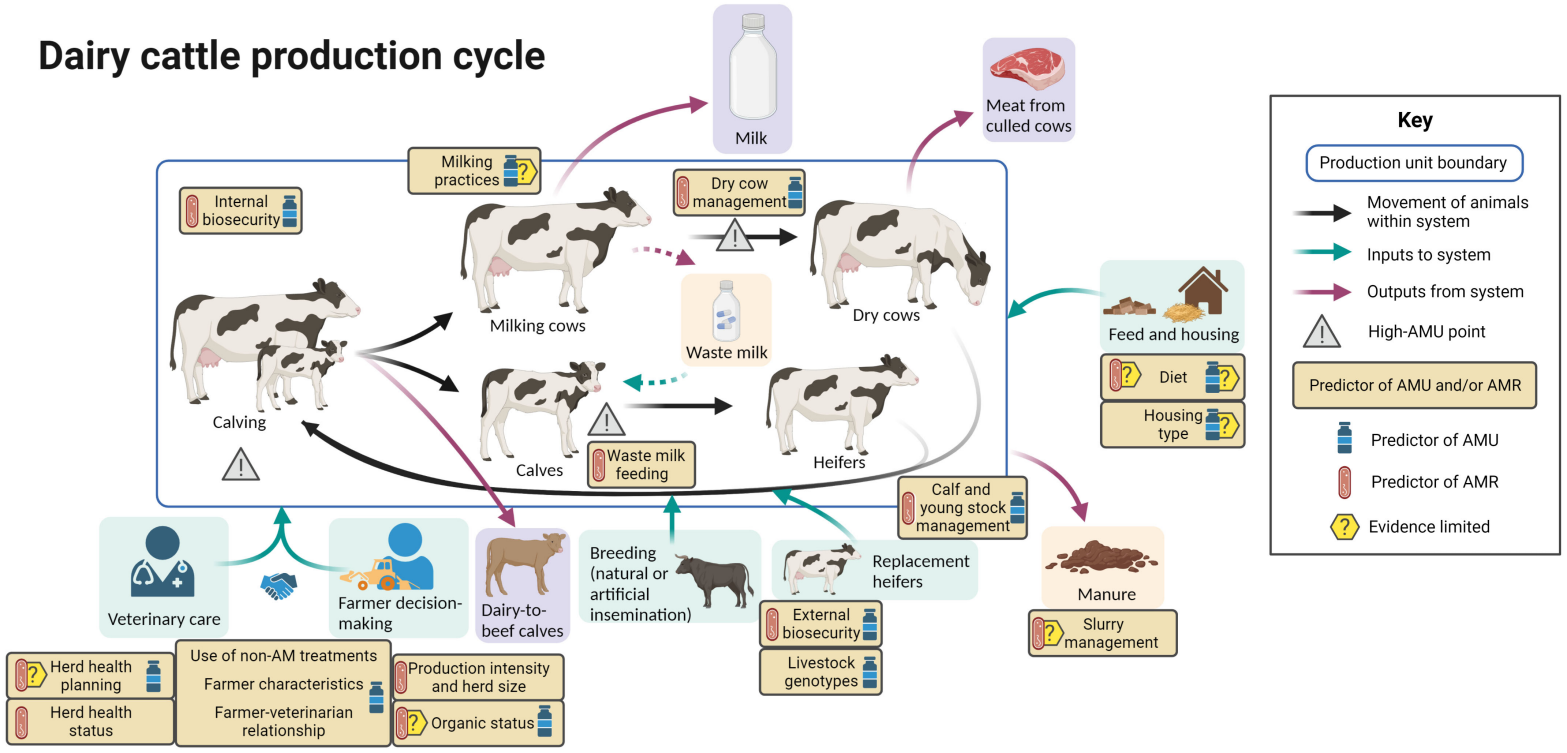
Factors associated with AMU and AMR in dairy cattle production. Created with BioRender.com.

**Figure 3** summarizes the evidence regarding factors associated with AMU and AMR at different points in the dairy cattle production cycle, including points in the cycle associated with increased AMU.

### 3.3 Beef cattle

In studies conducted in Europe, wide variations in AMU between different beef farms within each country have been observed (Responsible Use of Medicines in Agriculture Alliance (RUMA), 2021; Alliance to Save Our Antibiotics, 2021; Bos et al., 2013; Earley et al., 2019; Diana et al., 2021), suggesting that there is scope to reduce AMU markedly on some farms.

#### 3.3.1 Organic status

A pilot study of 119 beef farms suggests that in the UK, organic beef farms have lower AMU than conventional farms. As observed with broader UK beef sector data (Responsible Use of Medicines in Agriculture Alliance (RUMA), 2021), a small number of farms have disproportionately high AMU among this sample of organic farms (Alliance to Save Our Antibiotics, 2021).

#### 3.3.2 Farm characteristics

In Germany, larger herds had significantly higher odds of using any antimicrobials than smaller herds, potentially associated with buying in more stock (Hommerich et al., 2019). As well as overall numbers, two Italian studies suggest that cattle arriving on fattening farms in autumn or winter had a significantly higher AMU than those arriving in spring or summer (Diana et al., 2021; Santinello et al., 2022). Treatment incidence was significantly higher in male cattle (Diana et al., 2021), with sex-based differences in susceptibility to respiratory disease presented as a possible cause by the authors. This is consistent with the finding that a reduction in AMU associated with quarantine on arrival was significant for male but not female cattle (Santinello et al., 2022). Non-significant trends were also observed for higher AMU in Blonde d’Aquitaine and Limousin breed cattle (Diana et al., 2020; Diana et al., 2021). Factors characteristic of less intensive farming, including use of dual-purpose breeds, were significantly associated with lower prevalence of cefotaxime-resistant *E. coli* (Hille, 2017).

Beef production systems include calves from beef-only herds, and “dairy-to-beef” calves, which originate from dairy farms and may be dairy breeds or dairy crossed with beef. An analysis of AMU in Irish beef and dairy-to-beef calves across n=79 suckler beef farms and n=44 dairy farms found no significant difference in overall AMU between the two types of calves over the first 6 months of life but significant differences in indications for treatment. Beef-only farms showed significantly higher incidence of navel ill, joint ill and respiratory disease, while dairy-to-beef calves had a significantly higher incidence of diarrhea, which accounted for 58.3% of group treatments with antimicrobials (Earley et al., 2019). Beef cattle originating from dairy rather than beef-only farms had a significantly higher risk of harboring AMR *E. coli* in studies in Switzerland (Reist et al., 2013) and the UK (Velasova et al., 2019).

In the UK beef industry, calf-rearing units were associated with higher AMU than suckler or finisher herds (Responsible Use of Medicines in Agriculture Alliance (RUMA), 2021; Doidge et al., 2020). Mixing of calves from different farms after transport to a calf-rearing unit has been highlighted as a likely contributor due to the increased risk of pneumonia (Doidge et al., 2020) and the number of introduced calves was found to be the most important predictor of AMU in Danish veal and young bull production (Fertner et al., 2016). As well as higher AMU in younger age groups, beef calves demonstrated a higher risk of antimicrobial-resistant commensal bacteria than adult cattle (Reist et al., 2013), mirroring findings in dairy cattle (Reist et al., 2013).

#### 3.3.3 Biosecurity

Biosecurity was positively correlated with a higher composite management and welfare score based on farm management, staff training, housing, and animal-based measures in an Italian study. Higher composite scores were significantly associated with lower AMU, but biosecurity in itself was not; the authors attribute this to low biosecurity scores across their study population (Diana et al., 2020).

##### 3.3.3.1 Internal biosecurity

Dutch farms where veal stables were disinfected between every few production cycles as opposed to not at all had significantly lower AMU (Mallioris et al., 2021). Several hygiene practices such as fly control and indoor manure storage were significantly associated with lower prevalence of cefotaxime-resistant *E. coli* on beef cattle farms in Germany, but use of isolation pens for sick animals was associated with higher prevalence, possibly due to farms with higher disease prevalence being more likely to have dedicated sick pens (Hille, 2017).

##### 3.3.3.2 External biosecurity

An Italian trial found that a 30-day quarantine period for new arrivals on beef fattening farms significantly reduced the AMU for male cattle, although no significant difference was noted for female cattle (Santinello et al., 2022). In the UK, consideration of Johne’s disease when buying in new cattle was negatively associated with use of group antimicrobial treatments (Doidge et al., 2020).

Movement of cattle between farms was also identified as a risk factor for shedding of AMR bacteria in studies in Switzerland and the UK (Reist et al., 2013; Velasova et al., 2019). However, external biosecurity indicators such as open-herd policies and purchase rate of cattle were not significantly associated with AMR on German farms (Hille, 2017).

#### 3.3.4 Herd health

As noted above, significantly lower AMU was observed on farms with better composite animal management and welfare scores in beef fattening farms in Italy, but no conclusions could be made regarding the relative contributions of different components of this (Diana et al., 2020).

#### 3.3.5 Nutrition and housing

In Dutch veal systems, ambient temperatures above 10°C and provision of pelleted feed rather than other starter feeds were significantly associated with lower AMU, while straw bedding was associated with higher AMU (Mallioris et al., 2021). Multiblock analysis of the results of this study showed that housing microclimate explained the greatest part of the observed variation in AMU (Mallioris et al., 2021). In a UK study, beef cattle fed a majority-concentrate diet had a significantly higher diversity of antimicrobial genes and a broader spectrum of AMR mechanisms in the gut flora compared to those fed a diet consisting of equal amounts of forage and concentrate (Auffret et al., 2017).

#### 3.3.6 Human factors

Two UK studies of veterinarian and farmer decision-making found that the relationship between the farmer and their veterinarian influenced their AMU choices (Doidge et al., 2019; Doidge et al., 2020). Farmers tended to view veterinary advice very positively and articulated a desire for more veterinary guidance on disease prevention to facilitate reduced AMU (Doidge et al., 2020). The characteristics of the veterinarians themselves also predicted AMU decisions: veterinarians working in purely large animal practice, younger veterinarians and veterinarians in situations where practice colleagues had prescribed antimicrobials previously were all more likely to prescribe antimicrobials without a farm visit (Doidge et al., 2019). A 2017 survey of UK beef farmers identified knowledge gaps regarding HP-CIAs and reported that some respondents were unaware which of the veterinary medicines they were using were antimicrobials (Doidge et al., 2020). Use of digital farm management tools and movement records have been associated with lower AMU (Doidge et al., 2020) and lower AMR (Hille, 2017), potentially reflecting on broader farmer attitudes to farm management. As with dairy cattle, actual antimicrobial dosing in beef cattle has been observed to differ from recommended doses (Merle et al., 2014).

**Figure 4:**
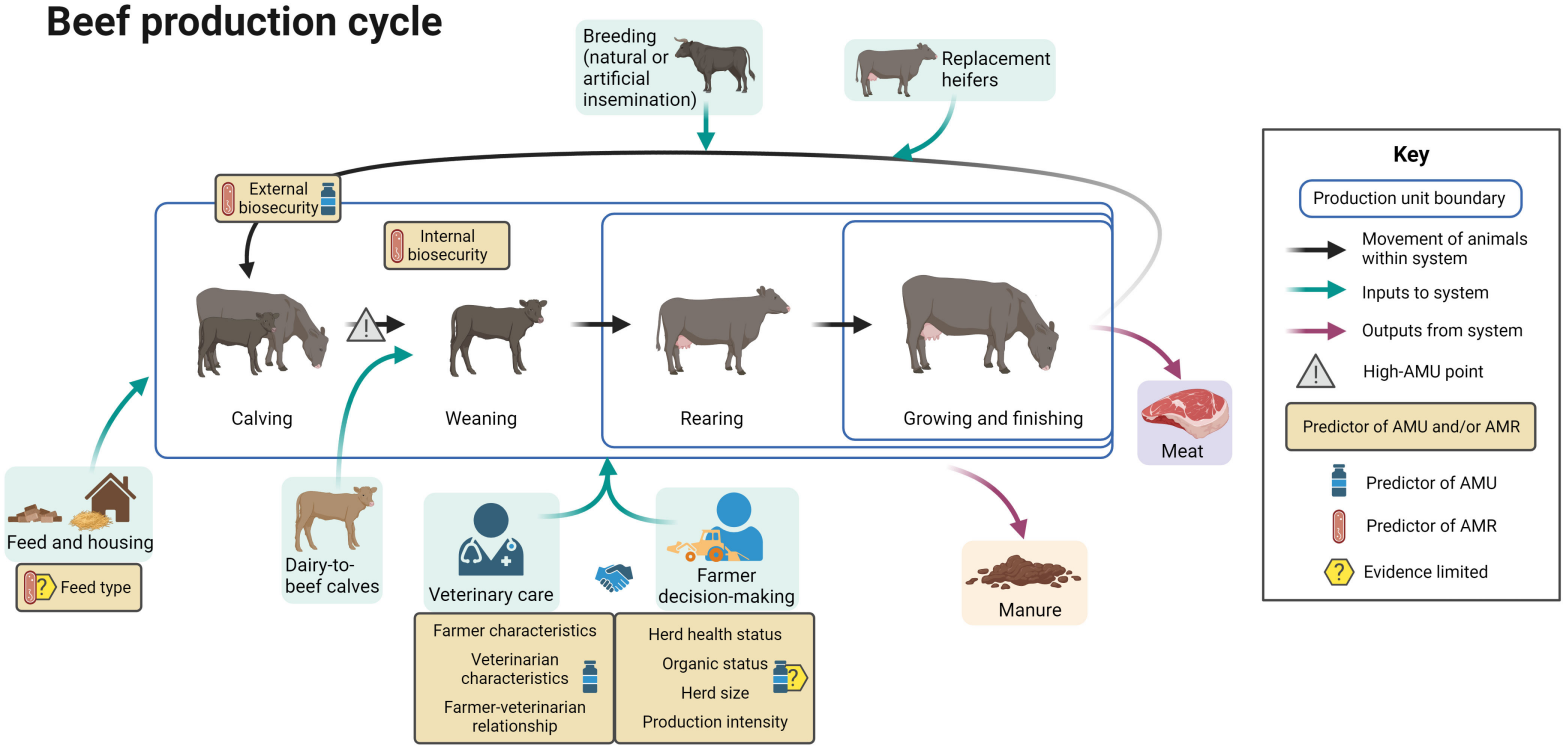
Factors associated with AMU and AMR in beef cattle production. Created with BioRender.com.

**Figure 4** summarizes the evidence regarding factors associated with AMU and AMR at different points in the beef production cycle, including points in the cycle associated with increased AMU. The differing boundaries of production units reflect the production stages included in each type of unit.

### 3.4 Sheep

As in other sectors, several UK studies have noted that small numbers of sheep farmers were disproportionately high users of antimicrobials both in the organic sector (Alliance to Save Our Antibiotics, 2021) and industry-wide (Davies et al., 2017; Lovatt and Davies, 2019; Responsible Use of Medicines in Agriculture Alliance (RUMA), 2019), suggesting a role for targeted interventions. No AMU figures using large sample populations were available and estimated mean AMU values given by academic studies varied from 11.48mg/kg to 16.7mg/kg, with some producer groups reporting mean AMU as low as 3.8mg/kg (Davies et al., 2017; Lovatt and Davies, 2019; Responsible Use of Medicines in Agriculture Alliance (RUMA), 2019). Literature addressing sheep was very limited and quantitative studies were identified only in the UK and Greece.

#### 3.4.1 Farm characteristics

A UK analysis found that lowland flocks had the highest AMU, followed by upland, then hill flocks (Davies et al., 2017), the reasons for which require further investigation. The same study identified a non-significant trend for lower AMU in organic flocks, consistent with the limited available industry data (Alliance to Save Our Antibiotics, 2021), although estimates for industry-wide AMU in sheep vary markedly. A study conducted in Greece reported a significantly lower prevalence of AMR in organic compared to conventionally raised dairy sheep (Malissiova et al., 2017), while another in Greece found a positive association between intensive production and presence of resistant *Staphylococcus* isolates in bulk milk tanks, although the criteria for intensive production were not specified (Lianou et al., 2021).

#### 3.4.2 Lambing practices

A UK study identified a possible association between the proportion of ewes lambing indoors and farm-level AMU, but this was not statistically significant (Lovatt and Davies, 2019).

#### 3.4.3 Biosecurity

The only study of biosecurity and AMU or AMR was an investigation of AMR in sheepdog puppies and lambs in Greece. This identified identical phenotypic resistance profiles in *E. coli* isolates from two pairs of animals (one puppy and one lamb) on the same farm (Chatzopoulos et al., 2016). Phylogenetic analysis would have been beneficial for further investigation.

#### 3.4.4 Herd health

No significant link was found between vaccination against footrot and AMU in a study of 152 UK flocks (Lovatt and Davies, 2019). In UK flocks, lameness accounted for 65.5% of the AMU in one study, and incidence varied markedly between farms (Davies et al., 2017), although the etiologies of the lameness cases were not differentiated.

#### 3.4.5 Human factors

A UK survey investigating uptake of contagious ovine digital dermatitis (CODD) management guidelines reported that 45% of veterinarians said that they decreased their use of whole-flock antimicrobial treatments and 57% were recommending reduction in use of antimicrobial footbaths on farms because of evidence-based guidelines. Farmers also reported changing their practices because of the updated advice, with 46% updating biosecurity measures and 52% reconsidering their choice of antibiotic (Duncan et al., 2022). As well as access to up-to-date guidelines, individual farmer characteristics, in particular attitudes to change, were identified as predictors of AMU (Doidge et al., 2021a).

Quantitative analysis of AMU on UK sheep farms reported that 21% of unexplained variation in farm AMU was between veterinary practices (Davies et al., 2017), suggesting differences in prescribing behaviors. A survey of veterinarians investigating prescribing decisions in various scenarios identified predictors including veterinarian characteristics as well as farmer-veterinarian relationships. Younger veterinarians and those working in farm-only veterinary practices were more likely to prescribe antimicrobials to a sheep farmer without a visit, while being confident in the farmer’s ability to identify the disease and feeling that the farmer was unwilling to pay for a visit also increased the likelihood of prescribing (Doidge et al., 2019).

**Figure 5:**
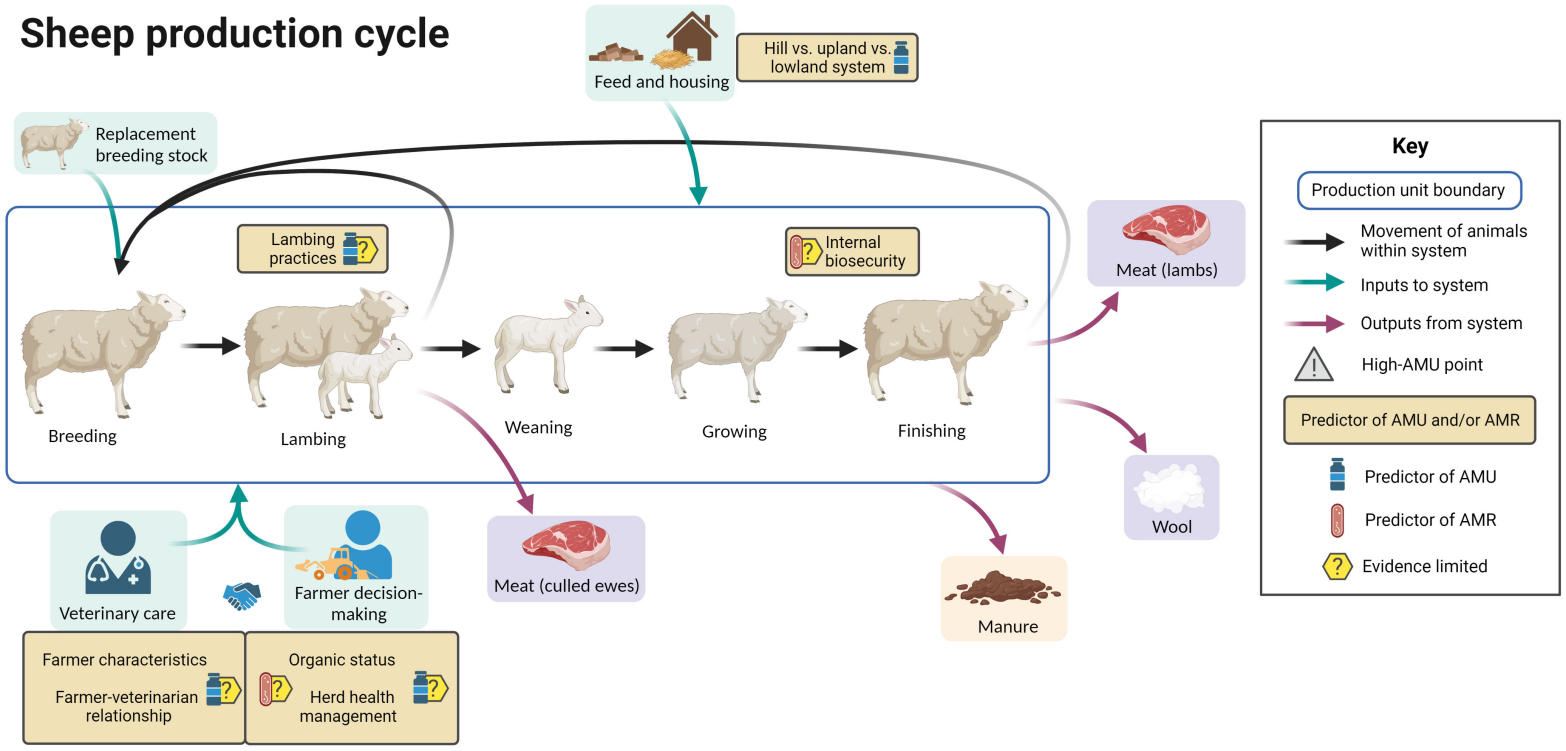
Factors associated with AMU and AMR in sheep production. Created with BioRender.com.

**Figure 5** summarizes the evidence regarding factors associated with AMU and AMR at different points in the sheep production cycle, including points in the cycle associated with increased AMU. Whilst sheep production in the UK is characterized by the movement of sheep from hill farms to upland to lowland farms, the production cycle is presented in a simplified manner here for clarity.

### 3.5 Pigs

Pig farming is a key focus for AMU and AMR research internationally. As with other species, pig farms show a large variation in AMU (Sarrazin et al., 2019), with a long-tailed distribution indicating a small number of farmers as high users (Van Der Fels-Klerx et al., 2011; Bos et al., 2013; Sjölund et al., 2015; Sjölund et al., 2016; Van Geijlswijk et al., 2019; Scali et al., 2020). A longitudinal study in the Netherlands found that the same farms continued to be high users year after year, rather than high usage in response to a specific disease outbreak (Van Der Fels-Klerx et al., 2011). This finding contrasts with the observation in UK dairy cattle that different herds were in the top quartile for AMU from year to year (Kingshay Independent Dairy Specialists, 2021).

#### 3.5.1 Organic status

A UK study carried out prior to the EU ban on AMU for growth promotion, which came into effect on 1 January 2006 (Casewell, 2003), observed markedly lower AMU on organic pig farms compared to conventional farms, although only 12 pig farms were included in the study (Veterinary Laboratories Agency (VLA), 2006). Recent data from Switzerland and the UK suggest this association between organic pig farming and lower AMU still exists, with a pilot study of 18 organic UK pig farms reporting mean AMU of 1.42mg/kg, compared to 110mg/kg reported in national monitoring (Alliance to Save Our Antibiotics, 2021; Arnold et al., 2016; Veterinary Medicines Directorate (VMD), 2020). In Denmark, AMU was significantly lower in the free-range herds than in the indoor herds, but there was no significant difference between organic free range and conventional free-range pigs (Nielsen et al., 2021). An association between membership of two farm assurance schemes (Scottish Pig Industry Initiative and Freedom Foods) and lower self-reported AMU was also observed in an older UK study, although AMU regulations and norms have changed significantly since the data for this study were collected in 2001 (Stevens et al., 2007). Taken together, these findings suggest that there is lower AMU in organic systems and free-range systems but that the differences between organic free-range and conventional free-range may not be as marked.

A cross-sectional study of conventional and organic pig farms in Denmark, France, Italy and Sweden, found a significantly higher prevalence of AMR amongst *E. coli* isolates from conventional rather than organically raised pigs in all four countries (Österberg et al., 2016). However, a fecal analysis covering the same four countries reported that organic status had little impact on antimicrobial resistance gene (ARG) abundance for the six ARGs measured, with country of origin the main predictor (Gerzova et al., 2015). Studies in the UK and the Netherlands have reported lower AMR prevalence on organic compared to conventional pig farms (Veterinary Laboratories Agency (VLA), 2006; Hoogenboom et al., 2008), although neither calculated p-values. Two Spanish studies found significant positive associations between intensive production and AMR when compared with organic and conventional free-range herds (Galan-Relano et al., 2019; Mencía-Ares et al., 2021).

AMU has been identified as a risk factor for AMR (Docic and Bilkei, 2003; Taylor et al., 2009; Burow et al., 2019; AbuOun et al., 2020; Ceccarelli et al., 2020; Luiken et al., 2020; Mencía-Ares et al., 2021; Luiken et al., 2022), although resistance is also observed in pigs with no history of antimicrobial treatment (Taylor et al., 2009; Burow et al., 2019) and one study noted a lack of correlation between AMU and AMR on 60 pig farms (De Koster et al., 2021). Specific aspects of treatment such as choice of antimicrobial or use of group treatments have been found to be significantly associated with AMR (Dohmen et al., 2017). Evidence that antimicrobial dosing rates used on farms frequently deviate from licensed or recommended doses (Merle et al., 2014; Sarrazin et al., 2019) highlights factors other than just quantity of antimicrobials used.

#### 3.5.2 Farm characteristics

Conflicting results were found regarding farm size as a determinant of AMU. Several studies found lower AMU in larger herds (Casal et al., 2007; Scali et al., 2020; Mallioris et al., 2021), some found the relationship with herd size non-significant (Van Rennings et al., 2015; Arnold et al., 2016; Backhans et al., 2016; Collineau et al., 2017a; Kruse et al., 2020), whilst others observed higher AMU in larger herds (Stevens et al., 2007; Van Der Fels-Klerx et al., 2011; Visschers et al., 2016; Collineau et al., 2018; Raasch et al., 2018; Stygar et al., 2020; Matheson et al., 2022). One possible reason that larger farms might have lower AMU is a positive association between farm size and level of biosecurity identified in several studies (Laanen et al., 2013; Mencía-Ares et al., 2021; Yun et al., 2021).

The production stages included in the farm appear to impact AMU and AMR, but the trends identified differ between studies. AMU was significantly higher in farrow-to-finish and piglet producers compared with finishing farms in Switzerland and England (Echtermann et al., 2019; Matheson et al., 2022), but the opposite trend was observed for antimicrobial prophylaxis in Spain (Casal et al., 2007). Other papers reported AMU to be higher in mixed-species farms compared with farrow-to-finish or specialized fattening farms (Van Der Fels-Klerx et al., 2011) and higher in farrow-to-finish than purely piglet producers (Van Der Fels-Klerx et al., 2011; Mallioris et al., 2021). In a UK study, finisher-only farms were at significantly higher risk of multi-drug resistant (MDR) *E. coli* presence than breeder-finisher farms, possibly due to the practice of buying pigs in from multiple sources (AbuOun et al., 2020).

#### 3.5.3 Key stages in production

Peaks in AMU were detected at weeks 1, 4 and 9, in pig farms in nine European countries, although relative contributions of therapeutic and prophylactic AMU to these peaks were not investigated (Sarrazin et al., 2019). These peaks coincide with the start of each production phase (piglet, weaner, finisher), which can involve stressful management changes and husbandry interventions.

Suckling pigs have been identified as a high-AMU age group (Van Rennings et al., 2015; Backhans et al., 2016; Raasch et al., 2018; Echtermann et al., 2019; Yun et al., 2021) but risk factors affecting relative AMU among piglets in this age group are uncertain. Surgical castration was found to have no effect on AMU in piglets in Spain despite impacts on productive performance (Morales et al., 2017), while tooth clipping in piglets showed a possible association with AMR in two studies but was not statistically significant in the multivariate models for either study (Dorado-García et al., 2015; Dohmen et al., 2017). Weaning is a period of high stress for piglets and is a period of particularly high risk for antimicrobial use (Sjölund et al., 2016; Martin et al., 2020; O'Neill et al., 2020), although relative AMU compared with suckling piglets varies between countries (Moreno, 2013; Sjölund et al., 2015). Later weaning age was significantly associated with lower AMU in one study of Dutch and Belgian pig farms (Caekebeke et al., 2020), while other studies either showed non-significant associations (Postma et al., 2016a), significance in only some of the countries covered (Collineau et al., 2018) or no association (Collineau et al., 2017a).

Whilst sows have been observed to have a lower prevalence of AMR commensals than piglets (Crombe et al., 2012) and other age groups (Nollet et al., 2006), similarities in resistance profiles of *E. coli* isolates from young piglets and their dams in Germany suggest colonization of piglets with maternal flora (Burow et al., 2019). In Spain, shedding of cephalosporin-resistant *E. coli* (CR-EC) by the dam was found to be the most important predictor for isolation of CR-EC in young pigs at slaughter, regardless of AMU history (Cameron-Veas et al., 2016).

#### 3.5.4 Biosecurity

Better overall biosecurity has been found to be significantly associated with decreased AMU (Laanen et al., 2013; Collineau et al., 2017a), but also with increased risk of AMR (Luiken et al., 2022), with these AMU and AMR findings also observed in studies addressing internal biosecurity specifically.

##### 3.5.4.1 Internal biosecurity

A wide range of studies have found associations between better internal biosecurity and reduced AMU (Laanen et al., 2013; Arnold et al., 2016; Postma et al., 2016b; Collineau et al., 2017a; Mallioris et al., 2021), although the statistical association was not always significant (Backhans et al., 2016; O'Neill et al., 2020; Mencía-Ares et al., 2021; Yun et al., 2021). In one case better internal biosecurity was associated with higher AMU, for which the authors were unable to offer any explanation (Raasch et al., 2018). Use of all-in-all-out (AIAO) rather than continuous production systems showed a possible association with reduced AMU in Switzerland (Arnold et al., 2016). Relatively long farrowing cycles, required for implementation of a strict AIAO system, were associated with lower AMU in data covering Belgium, France, Germany and Sweden (Postma et al., 2016a; Collineau et al., 2018). Practices avoiding unnecessary mixing of animals, for example maintaining stable sow groups (Dorado-García et al., 2015) and using an AIAO rather than continuous production system, were associated with lower AMR (Schuppers et al., 2005) in the Netherlands and Switzerland respectively.

Consistent with the counterintuitive findings for overall biosecurity, widely considered a protective factor, a study of fecal resistomes from pigs in nine European countries found a significant positive association between higher standardized internal biosecurity scores and presence of macrolide resistance genes (Van Gompel et al., 2019). In a German study, the presence of a hospital pen on the farm and use of chemical fly control were both associated with increased risk of AMR (Hering et al., 2014). However, cleaning practices were associated with reduced AMR in other studies in pigs in the UK and Netherlands (Dorado-García et al., 2015; AbuOun et al., 2020), as was improved manure management in the UK (AbuOun et al., 2020).

Presence of other species on the farm has been identified as a possible risk factor for AMR transmission, with pigs acting as either the source or recipient of the resistant flora, with similarities in MRSA lineages from cattle, humans and pigs on Italian farms, suggestive of transfer between species (Locatelli et al., 2017). A Dutch study found the presence of goats on the same farm to be significantly positively associated with ESBL *E. coli* presence in pigs (Dohmen et al., 2017). In Belgium, the presence of dairy cattle or chickens was not a significant risk factor for MRSA carriage in pigs, but pigs were identified as a possible reservoir of MRSA and were significantly more likely to carry MRSA than cattle or poultry on the same farms (Verhegghe et al., 2013). However, a UK study found that being based within a mile of a poultry farm was negatively associated with AMR presence (Taylor et al., 2009) and genomic analysis of pigs and broilers across Europe showed that the two species had distinct resistomes (Munk et al., 2018).

##### 3.5.4.2 External biosecurity

External biosecurity has been identified as a significant protective factor against AMU in numerous studies (Casal et al., 2007; Arnold et al., 2016; Postma et al., 2016a; Postma et al., 2016b; Raasch et al., 2018), although not all studies found the association to be significant (O'Neill et al., 2020; Mencía-Ares et al., 2021).

Open pig farms were found to be associated with increased AMU compared to closed herds in the Netherlands (Dorado-García et al., 2015; Dohmen et al., 2017), although not every study found a significant association (Mallioris et al., 2021). In the Netherlands, the UK and Belgium, significant positive associations were found between AMR and open herds (Taylor et al., 2009; Crombe et al., 2012; Dorado-García et al., 2015). On Dutch pig farms, biosecurity measures focusing on workers and supplies were associated with reduced risk of ESBL *E. coli* (Dohmen et al., 2017) and MRSA (Dorado-García et al., 2015).

A genomic analysis of quinolone-resistant *E. coli* (QREC) isolates in Norway identified two closely-related isolates from pigs in different parts of the country (Kaspersen et al., 2020). The authors suggest that this may reflect dissemination of resistant bacteria down the pig supply chain. Local spread of pathogens, as well as resistant bacteria, between pigs is hypothesized to result in an increased risk of AMU (Van Der Fels-Klerx et al., 2011; Arnold et al., 2016; Collineau et al., 2017a; Raasch et al., 2018) and AMR (Taylor et al., 2009; AbuOun et al., 2020; Luiken et al., 2022) in herds kept in high-density pig farming areas. In a UK study, farms that required visitors to be free of pig contact for at least two days before arrival on-farm had significantly lower AMR risk (Taylor et al., 2009).

#### 3.5.5 Herd health

Use of homeopathic treatments had a significant negative association with AMU on Swiss fattening farms (Arnold et al., 2016), while use of group treatments for antimicrobials was associated with a higher overall AMU (Casal et al., 2007; Merle et al., 2014; Martin et al., 2020). General good health of the animals on-farm and use of anthelmintics were negatively associated with AMR in Swiss finishing farms (Schuppers et al., 2005).

In Swedish pig herds, weaners from specific pathogen-free herds had lower AMU than those from other herds (Sjölund et al., 2015). In Denmark, AMU to treat respiratory signs was found to be closely associated with seroconversion to porcine respiratory disease complex (PRDC) (Antunes et al., 2019), suggesting that PRDC control may contribute to minimizing AMU.

Several studies found significant associations between vaccination and lower AMU, although these associations were limited either to use of multiple vaccinations (Collineau et al., 2017a) or introduction of vaccines to address existing problems (Fricke et al., 2015; Rojo-Gimeno et al., 2016). Several other papers reported no significant changes in AMU with various vaccination protocols (Sjölund et al., 2015; Kruse et al., 2017; Mallioris et al., 2021). Some vaccinations were associated with higher AMU (Stevens et al., 2007; Postma et al., 2016a; Collineau et al., 2018; O'Neill et al., 2020) and one had a significant positive association with MRSA risk (Dorado-García et al., 2015).

#### 3.5.6 Nutrition and water

Use of a private water source rather than mains has been identified as a risk factor for increased AMU on Dutch farms, potentially due to inconsistent water quality (Mallioris et al., 2021). The practice of farms milling their own feed was also significantly associated with reduced AMU in a study of Irish pig farms, although the authors point out that this may be a biosecurity indicator (O'Neill et al., 2020). Links between feeding practices and AMR were identified, with feeding of whey and limited (rather than ad libitum) feeding significantly negatively associated with AMR in Swiss finishers (Schuppers et al., 2005).

Feed composition may influence the livestock resistome, mediated by nutritional effects on the gut microbiome. Taxonomic variation in the gut microbiome was found to explain resistome variation in pigs on farms across Europe, suggesting that by influencing the gut microbiota, it may be possible to limit colonization by resistant organisms (Munk et al., 2018).

Marketing authorization for medicinal products containing zinc oxide at therapeutic levels (≥1500ppm) was withdrawn in the EU in June 2022, following a 5-year phasing out period, in order to reduce selection pressure for AMR associated with zinc supplementation. One study found no significant association between in-feed zinc and AMU (Sjölund et al., 2015), while another found a non-significant trend of positive correlation between AMU and zinc use (Nielsen et al., 2021). In a controlled trial, in-feed zinc supplementation at 2500 ppm was significantly associated with MDR *E. coli* carriage (Bednorz et al., 2013).

#### 3.5.7 Housing

An experimental comparison of fully-slatted and straw-bedded floors reported overall reduced AMU on fully-slatted rather than straw-bedded floors (Scott et al., 2006). On UK pig farms, having finisher pens with outdoor space and use of automatically controlled natural ventilation were both associated with lower AMU (Matheson et al., 2022). Finnish fattening farms with average-to-poor air quality or problems with pen cleanliness and condition were found to have higher AMU, and a significant positive interaction effect was observed between high stocking density and poor pen condition (Stygar et al., 2020). A study covering nine European countries found high pig stocking density to be positively associated with AMR genes in the farm environment (Luiken et al., 2022).

#### 3.5.8 Human factors

Evidence of the role of socio-demographic characteristics as predictors of AMU appears inconsistent. One study found higher AMU in farms run by older farmers (Backhans et al., 2016), while others found no significant association between age and AMU (Postma et al., 2016a; Visschers et al., 2016). Farmer gender was not significant in most studies (Postma et al., 2016a; Visschers et al., 2016; Collineau et al., 2017a), although one study found female farmers significantly associated with higher AMU in suckling piglets (Backhans et al., 2016). The same study suggested university-educated farmers reported significantly higher AMU in suckling piglets, but the association between education and AMU was not identified in another study the same year (Postma et al., 2016b). In a study of veterinary prescribing on German pig farms, there was significant variation between individual veterinarians’ prescribing practices but predictors were not explored (Van Rennings et al., 2015).

Use of external farm consultant services was associated with lower AMU in Swiss farms, potentially an indicator of farmer attitudes or consultant advice (Arnold et al., 2016). General views regarding antimicrobials were found to be a significant predictor of AMU among Swiss (Visschers et al., 2014) but not Swedish farmers (Backhans et al., 2016). Perceiving an expectation from society to reduce AMU and scoring higher for optimistic views regarding the risks and benefits of reducing AMU were both associated with lower AMU by farmers (Van Asseldonk et al., 2020).

While financial concerns were reported as a key barrier to reducing AMU (Visschers et al., 2014; Van Asseldonk et al., 2020), early trials of farm-specific AMU reduction plans showed a net economic gain for farmers (Rojo-Gimeno et al., 2016) and have been demonstrated to be successful in reducing AMU without compromising production parameters (Rojo-Gimeno et al., 2016; Collineau et al., 2017b). Voluntary schemes and mandatory national programs involving external monitoring of farm AMU were associated with AMU reductions in Switzerland (Echtermann et al., 2020) and Denmark (Jensen et al., 2014) respectively.

**Figure 6:**
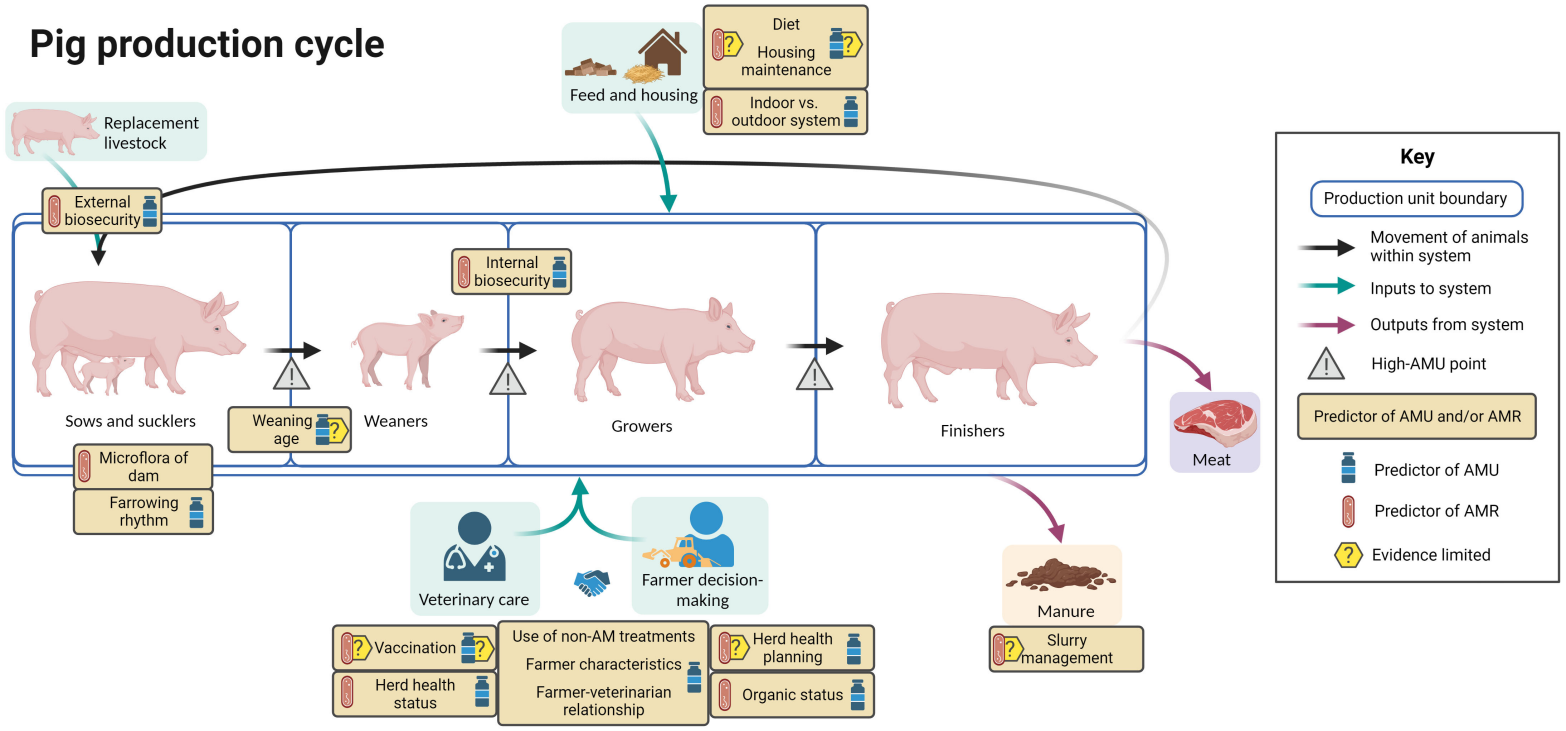
Factors associated with AMU and AMR in pig production. Created with BioRender.com.

**Figure 6** summarizes the evidence regarding factors associated with AMU and AMR at different points in the pig production cycle, including points in the cycle associated with increased AMU. In Europe, unit types include multiple combinations of production stage. Breeder farms breed piglets to sell as piglets or weaners or to rear to slaughter. Nursery units buy in piglets to sell on as weaners or to rear to slaughter, while finisher farms buy in weaners to rear to slaughter.

### 3.6 Broiler chickens

As in other species, great variation in AMU was observed between broiler farms, including among organic broiler farms (Alliance to Save Our Antibiotics, 2021; Persoons et al., 2012; Bos et al., 2013). Dutch broiler farms reported mean consumption of 23.8 animal treatment days per year (ATD/Y), with a standard deviation of 23.8 ATD/Y and some farms using over 140 ATD/Y (Bos et al., 2013). Some farms were found to achieve high technical performance with low AMU (Roskam et al., 2020) or operate intensive broiler production with no AMU during the study period (Persoons et al., 2012; Joosten et al., 2019; Luiken et al., 2019; de Jong and van Riel, 2020). AMU has been found to be positively associated with AMR in broiler chickens (Ceccarelli et al., 2020; Luiken et al., 2020) but sometimes the effect was small (Luiken et al., 2019) or the association non-significant (Luiken et al., 2022).

#### 3.6.1 Organic status

A UK study reported that of the seven organic broiler farms visited, only one had used antimicrobials in the two-year period examined, compared to all of the six conventional farms, although this was prior to the 2006 EU ban on AMU for growth promotion (Veterinary Laboratories Agency (VLA), 2006). A more recent UK report using a sample of six organic broiler farms gave a substantially lower mean AMU, at 2.95mg/kg (Alliance to Save Our Antibiotics, 2021) than the industry average of 17mg/kg PCU (Veterinary Medicines Directorate (VMD), 2020).

AMR patterns among organic and conventional farms have been much more widely researched and consistently reported lower AMR in chickens from organic farms (Soonthornchaikul et al., 2006; Hoogenboom et al., 2008; Miranda et al., 2008; Alvarez-Fernandez et al., 2013; Much et al., 2019; Hansson et al., 2021). However, some papers also identified a higher bacterial load in organic chickens (Hoogenboom et al., 2008; Miranda et al., 2008; Alvarez-Fernandez et al., 2013) and resistance patterns for individual antimicrobials were sometimes less clear (Miranda et al., 2008; Much et al., 2019). In Italy, antibiotic-free intensive broiler production was associated with significantly lower AMR in commensal flora than conventional production (De Cesare et al., 2022), although in this and other studies, AMR bacteria were detected despite absence of AMU (Veterinary Laboratories Agency (VLA), 2006).

#### 3.6.2 Farm characteristics

Dutch national statistics reported higher AMU in breeding flocks than in fattening flocks (Van Geijlswijk et al., 2019). In Irish farms, nearly half of all AMU in broilers was reported to be in the first week of life (Martin et al., 2020). Fattening farm traits associated with higher AMU included proximity to other farms (Caucci et al., 2019), higher broiler weight at slaughter (Hughes et al., 2008), and, possibly, larger farms (Bos et al., 2013). An analysis of different stages in the broiler production chain found that variations in AMU on broiler fattening farms were not affected by the breeder from which the chicks were sourced. AMU differences were largely associated with the different fattening farms as well as unexplained variation between chick batches (de Jong and van Riel, 2020).

#### 3.6.3 Indoor and free-range systems

Although Dutch national statistics reported lower AMU in farms with “alternative” chicken breeds associated with less intensive systems (Van Geijlswijk et al., 2019), an experimental study of fast- and slow-growing broiler breeds with no antimicrobial treatment showed a similarly high rate of AMR in both groups (Montoro-Dasi et al., 2020). The same authors observed rapid development of resistance without any AMU in another experiment, and found no significant differences in AMR between groups with high and low stocking density or groups with different levels of ventilation (Montoro-Dasi et al., 2021).

One study of intensive, semi-intensive and organic free-range systems in Portugal found significantly lower AMR in broilers from semi-intensive farms (described as “indoor extensive” in the study) than from either intensive or organic systems (Fraqueza et al., 2014). Another comparing intensive and extensive systems found no significant differences in AMR profiles between systems (Oliveira et al., 2010). Similarly, no significant difference was found between AMR prevalence in free-range and housed chickens in Greece (Economou et al., 2015). A significantly higher prevalence of tetracycline resistance was found in indoor than free-range chickens in a French study, but this could be due to the fact that in this study, carried out prior to the phasing out of AGPs in Europe, around half of the indoor farms and none of the free-range farms reported using AGPs (Avrain et al., 2003).

#### 3.6.4 Biosecurity

Possible links between lower AMU and better overall biosecurity were identified in a Belgian-Dutch study (Caekebeke et al., 2020). Multinational metagenomic studies gave mixed results: one found little evidence for any impact of biosecurity on broiler fecal resistomes (Luiken et al., 2019), but another found a significant negative association between better biosecurity and relative AMR gene (ARG) abundance in dust from broiler houses (Luiken et al., 2022).

##### 3.6.4.1 Internal biosecurity

Two studies in Germany found evidence of infection of chicks with AMR bacteria at the hatchery stage, despite disinfection of eggs (Projahn et al., 2017; Daehre et al., 2018a). Further down the production pyramid, studies in several countries found evidence of resistant bacteria persisting in broiler houses between flocks (Persoons et al., 2010; Börjesson et al., 2016; Daehre et al., 2018b) and that disinfection between production cycles reduced AMR risk (Mo et al., 2016). Although in several cases, the findings could also be explained by dissemination of AMR bacteria through the broiler production chain (Persoons et al., 2010; Daehre et al., 2018b). Transmission between species on mixed-species farms may also contribute to AMR in broilers. Clonal relationships between MRSA isolates in pigs and chickens have been identified (Verhegghe et al., 2013). However, a metagenomic analysis found a significant positive association between internal biosecurity score and oxazolidinone resistance genes, even after controlling for AMU (Luiken et al., 2019).

##### 3.6.4.2 External biosecurity

The practice of sourcing chicks from more than one hatchery was identified as a predictor of increased AMU for disease prevention and decreased AMU for disease treatment in UK broiler farms, although the study did not report the net difference in AMU (Hughes et al., 2008). In Norway, farms sourcing day-old broiler chicks from three or more parent flocks were at significantly higher risk of AMR, as were farms with less strict visitor biosecurity (Mo et al., 2016).

##### 3.6.4.3 Persistence of AMR in production chains

Several investigations have found evidence of resistant organisms persisting in parent and grandparent flocks and infecting flocks further down the production pyramid in Norway, Sweden, Denmark and Germany (Agersø et al., 2014; Mo et al., 2014; Börjesson et al., 2016; Projahn et al., 2017; Daehre et al., 2018a; Daehre et al., 2018b; Kaspersen et al., 2020) and this apparent pseudo-vertical transmission has been proposed as a possible explanation for lack of correlation between AMU and AMR (De Koster et al., 2021).

#### 3.6.5 Nutrition and housing

A study of French free-range broilers identified the use of chicken paper topped with feed to be a protective factor for AMU in hatchlings (Adam et al., 2019). No significant effect of stocking density on AMU was found on conventional farms in Italy (Tarakdjian et al., 2020). The impacts of bedding materials are unclear. Thinner litter was associated with lower AMU in French broilers, although the reasons for this were not clear (Adam et al., 2019). A metagenomic study found mixed results regarding the effects of bedding on ARGs in the environment. Shredded straw bedding was associated with lower absolute abundance of ARGs but higher relative abundance of ARGs compared to the environmental bacterial population. The same study found lower absolute ARG abundance during summer, which could be suggestive of climatic influences (Luiken et al., 2022).

In the UK, controlled (rather than *ad libitum*) feeding and use of competitive exclusion (CE) products were both negatively associated with prophylactic AMU (Hughes et al., 2008). As well as associations with AMU, laboratory trials have shown some promise for CE products to mitigate AMR (Ceccarelli et al., 2017; Dame-Korevaar et al., 2020; Methner and Rösler, 2020). A genomic study across nine European countries found that taxonomic variation in gut microbiome, potentially influenced by diet, explained resistome variation in broilers (Munk et al., 2018).

#### 3.6.6 Flock health

Therapeutic AMU on UK broiler farms was reported mainly in response to mortality, respiratory or enteric disease (especially necrotic enteritis) (Hughes et al., 2008), similar to other countries in Europe, although AMU for respiratory disease varies greatly between countries (Joosten et al., 2019). In UK broilers, vaccination against infectious bursal disease was positively associated with higher therapeutic AMU (Hughes et al., 2008), potentially due to temporary immunosuppression caused by this vaccine (Prandini et al., 2016). In free-range broilers in France, the use of essential oils as prophylactic treatment for any condition was significantly associated with lower AMU (Adam et al., 2019).

#### 3.6.7 Human factors

Farmer and veterinarian attitudes have not been as thoroughly researched for broiler chickens as for some other livestock species. As in other species, dosing accuracy may vary (Persoons et al., 2012). In France, AMU in broilers was significantly associated with the farmer’s perception of their flock’s health (Adam et al., 2019). A European trial of interventions targeting farmer behavior, along with general farm management and disease prevention, reported successes in reducing broiler farm AMU without negatively impacting economic performance (Roskam et al., 2019).

**Figure 7:**
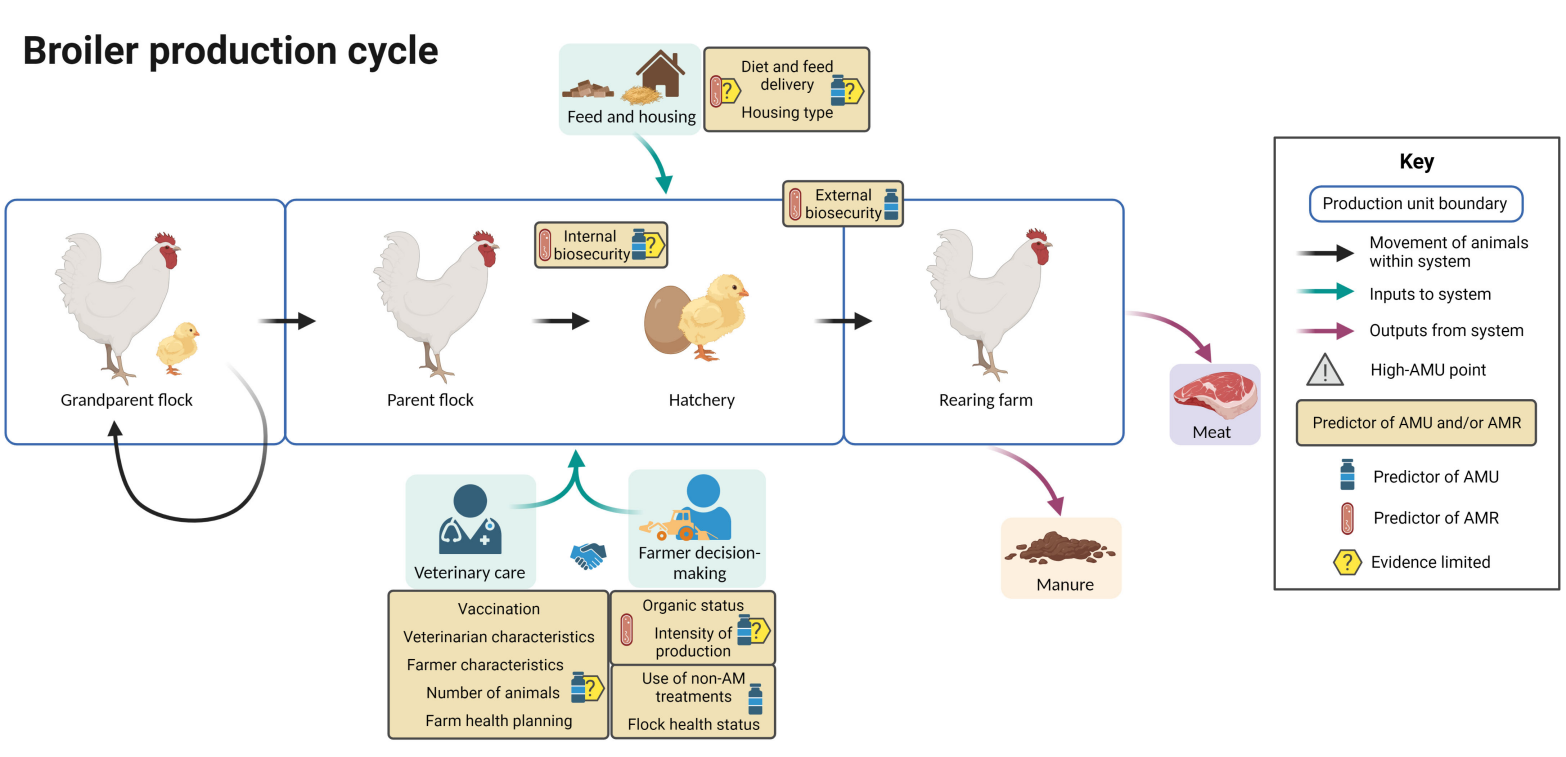
Factors associated with AMU and AMR in broiler chicken production. Created with BioRender.com.

**Figure 7** summarizes the evidence regarding factors associated with AMU and AMR at different points in the broiler production cycle, including points in the cycle associated with increased AMU. In broiler chickens, grandparent, parent and fattening units in the same production chain may be spread across different countries.

### 3.7 Laying chickens

Although data are limited, AMU in laying hens appears to show substantial variation, with Dutch national monitoring reporting a small number of relatively high-AMU farms (Van Geijlswijk et al., 2019). A 2011 study of laying hens across four European countries, reported zero AMU for over 90% of the flocks during the study period (Van Hoorebeke et al., 2011).

#### 3.7.1 Organic status

No significant difference in probability of AMU was found between organic free-range and battery hens across Belgium, Germany, Italy, and Switzerland (Van Hoorebeke et al., 2011). Although a sample of 14 UK organic laying hen farms reported lower AMU than the industry mean in 2018-2019 (Alliance to Save Our Antibiotics, 2021; Veterinary Medicines Directorate (VMD), 2020), further studies are required to substantiate this.

A pair of analyses comparing organic and conventionally reared laying hens in Germany identified significantly lower AMR prevalence in *E. coli* and *Enterococcus* species in organic chickens although a less clear-cut pattern was observed for *Campylobacter jejuni* (Schwaiger et al., 2008; Schwaiger et al., 2010) and a further study found no significant difference in AMR risk between organic free-range and conventional battery hens (Van Hoorebeke et al., 2011).

#### 3.7.2 Farm characteristics

National level data from the Netherlands reported AMU in rearing farms for laying hens to be substantially higher than laying hen farms (Van Geijlswijk et al., 2019), but no studies were found investigating AMR at different production stages. Contact with other species may be an AMR risk: in Belgian mixed-species farms with MRSA-positive pigs, MRSA was also isolated from chickens, albeit at a lower prevalence (Verhegghe et al., 2013).

In a survey of Scottish backyard poultry keepers, over 60% of respondents reported no AMU, and those who did report using any antimicrobials administered them less than once a year, although equivalent statistics are not available for commercial flocks to aid comparison. Inconsistent biosecurity and infrequent veterinary attention were highlighted by the authors as a risk for spread of AMR and infectious disease in general (Correia-Gomes and Sparks, 2020). Another UK study found that backyard poultry treated in companion animal veterinary practices often had advanced disease when presented at the practice. In 33.0% of chicken consultations, antimicrobials were prescribed, and 43.8% of these were HP-CIAs (Singleton et al., 2021).

#### 3.7.3 Nutrition and housing

A UK supermarket publishing AMU figures in 2018 reported higher AMU on its free-range egg supplier farms compared with cage or colony egg suppliers, but did not provide methodology or analysis (ASDA, 2018). No significant difference in probability of using any antimicrobials was found between different housing types in four European countries. This study did find some effects of housing type on AMR, with resistance in *E. faecalis* lower in free-range conventional farms compared to caged battery farms, but higher in *E. coli* in farms using raised-floor housing compared to battery cages (Van Hoorebeke et al., 2011). An observational study of laying hen farms in Switzerland found no consistent association between housing and management factors and AMR (Harisberger et al., 2011).

No publications were identified investigating diet or supplements, although laboratory-based studies have suggested that administration of CE flora in early life may protect against colonization with AMR *E. coli* (Methner et al., 2019; Methner and Rösler, 2020).

#### 3.7.4 Human factors

In a survey of UK free-range egg farmers, sourcing medications from veterinarians rather than agricultural merchants was significantly associated with introducing AMU reduction measures on their farms and increased contact with veterinarians was associated with a higher level of optimism about the scope for AMU reduction (Rayner et al., 2019). It is worth noting that in the UK antibiotics require a veterinary prescription, while some non-antibiotic medications can be bought without prescriptions.

**Figure 8:**
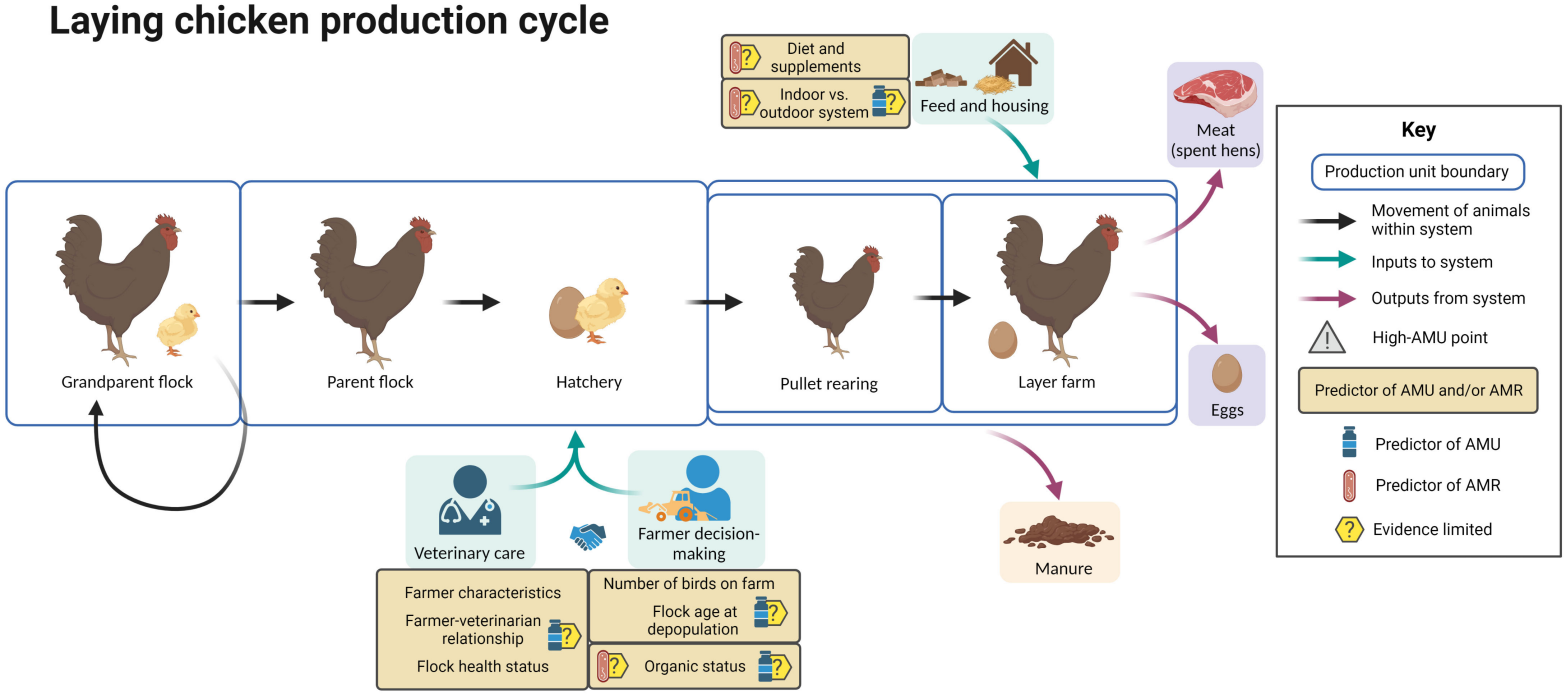
Factors associated with AMU and AMR in laying chicken production. Created with BioRender.com.

**Figure 8** summarizes the available evidence regarding factors associated with AMU and AMR at different points in the laying chicken production cycle.

### 3.8 Broiler turkeys

National monitoring in European countries indicates higher AMU per unit biomass in turkeys compared to other meat poultry species (Van Geijlswijk et al., 2019; Veterinary Medicines Directorate (VMD), 2022), but beyond this, data are limited.

#### 3.8.1 Organic status

Apparently no studies have compared AMU between representative samples of organic and conventional turkey farms. In a report on UK organic farms covering different livestock types, only one organic turkey farm (which recorded no antimicrobial usage) had responded to the request for data (Alliance to Save Our Antibiotics, 2021). An Italian study comparing AMR and natural immunity in organic and conventional turkey farms reported a general tendency for lower AMU in organic farms but reported no statistical analysis of the trend. In this study organic farms, which had stricter requirements for welfare standards such as stocking density, showed a non-significant trend for lower AMR. Organic status was also significantly associated with higher serum lysozyme concentration and serum bactericidal activity, indicators of natural immunity, which the authors attribute to better welfare, and which they suggest may contribute to lower AMR (Mughini-Gras et al., 2020). Comparison of organically and conventionally produced turkey meat in Germany found significantly lower AMR prevalence in organically produced meat but higher prevalence of *Campylobacter* (Tenhagen et al., 2020). As with other livestock species, AMR has been identified in turkeys that have not been treated with antimicrobials at all (Horie et al., 2021). Two studies, one a prevalence survey of phenotypic resistance in turkey commensal *E. coli* and the other a metagenomic analysis of turkey fecal resistomes, found no significant association between AMU and AMR in three European countries (Horie et al., 2021; Ceccarelli et al., 2020).

#### 3.8.2 Farm characteristics

Among Italian turkey farms, being located in an area with a higher density of turkey farms was a significant risk factor for higher AMU (Caucci et al., 2019). In the UK, turkey breeding farms with over 10,000 birds had a significantly higher risk of AMR presence than farms with fewer birds, but this pattern was not seen in fattening turkey farms (Jones et al., 2013).

#### 3.8.3 Biosecurity

Compliance with biosecurity protocols on French turkey farms, such as changing clothes and shoes before entering the facility, were found to be a significant protective factor for AMU (Chauvin et al., 2005). In UK turkey farms, both internal and external biosecurity factors were significantly associated with risk of AMR. Factors indicating contact with, or proximity to, other animal species were significant risk factors for AMR, while good hygiene and disinfection practices were significant protective factors (Jones et al., 2013). In contrast, a study of turkey farms in Germany, France and Spain found no significant associations with biosecurity but a significant negative association between proximity to another turkey farm and presence of AMR *E. coli* (Horie et al., 2021). Biosecurity at hatchery level was identified as an important control point by a UK study which found resistant *E. coli* and *Salmonella* in multiple areas of the hatchery environment, and evidence of pseudo-vertical transmission even in hatcheries using the recommended egg collection and disinfection protocols (Mueller-Doblies et al., 2013).

#### 3.8.4 Flock health

In fattening turkeys in France, use of CE flora was significantly associated with lower AMU. In this study, farmers having expectations of veterinary antimicrobial prescriptions and these expectations being met were significantly associated with higher overall AMU compared to farms in which staff had no particular expectations of antimicrobial prescriptions (Chauvin et al., 2005).

**Figure 9:**
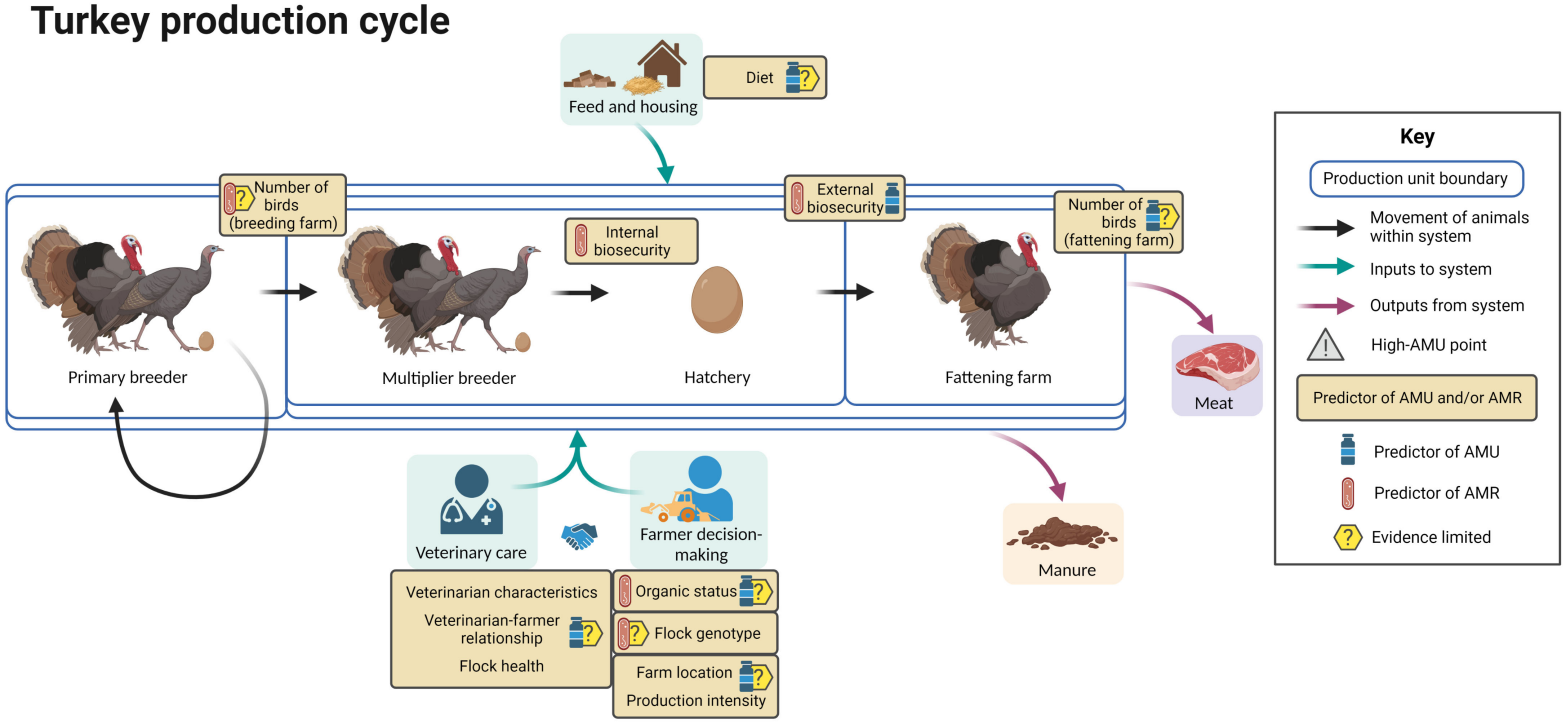
Factors associated with AMU and AMR in broiler turkey production. Created with BioRender.com.

**Figure 9** summarizes the available evidence regarding factors associated with AMU and AMR at different points in the broiler turkey production cycle.

### 3.9 Salmon

No studies presenting data on risk factors for AMU and AMR on salmon farms were found. Recently released data from the Scottish Environmental Protection Agency (SEPA) indicate a trend for increased AMU in Scottish salmon farms, with a small number of farms accounting for a large increase (Scottish Environmental Protection Agency (SEPA), 2022). The two antimicrobials used, florfenicol and oxytetracycline, are both licensed in salmon for treatment of furunculosis (caused by *Aeromonas salmonicida*), which has been reported as the main indication for increased AMU. However, financial reporting by the company using the greatest mass of antimicrobials reported that in 2021 their Scottish salmon farms had also experienced problems with several viral diseases, amoebic gill disease and sea lice (MOWI, 2022).

Vaccination was the only salmon management factor for which peer-reviewed studies in Europe were identified. Temporal AMU trends in Norwegian aquaculture supported the role of vaccines as a key aspect of limiting the need for antimicrobials. Furunculosis was the major indication for salmon AMU in the early 1990s, but after vaccination against this disease was introduced in 1992, there were almost no antimicrobials prescribed for furunculosis from the following year onwards, with overall antimicrobial use in aquaculture dropping dramatically (Lillehaug et al., 2003). Cold-water vibriosis is another bacterial disease controlled by vaccination, and a brief epidemic in Norway – attributed by the authors to changes in vaccination – coincided with a peak in the national levels of antimicrobial use in aquaculture, with a dramatic rise in the number of antimicrobial prescriptions for this disease (Lillehaug et al., 2018). In 2021, 0.58 mg/kg PCU of antimicrobials were used in Norwegian aquaculture (NIPC and T.N.V. Institute, 2021) compared to 41.3mg/kg PCU in the UK (Veterinary Medicines Directorate (VMD), 2022). Vaccination is presently carried out in 100% of farmed Scottish salmon before the start of the seawater phase of production (Responsible Use of Medicines in Agriculture Alliance (RUMA), 2019), suggesting that factors other than vaccination account for differences between AMU in the Norwegian and Scottish salmon industries.

Staffing and market conditions, affected by external situations such as COVID-19, may also affect AMU. An industry report attributed the rise in Scottish salmon AMU in 2020 at least in part to the larger biomass remaining on farms during the pandemic (Responsible Use of Medicines in Agriculture Alliance (RUMA), 2021), although as previously noted, AMU was increasing prior to the pandemic (Scottish Environmental Protection Agency (SEPA), 2022).

**Figure 10:**
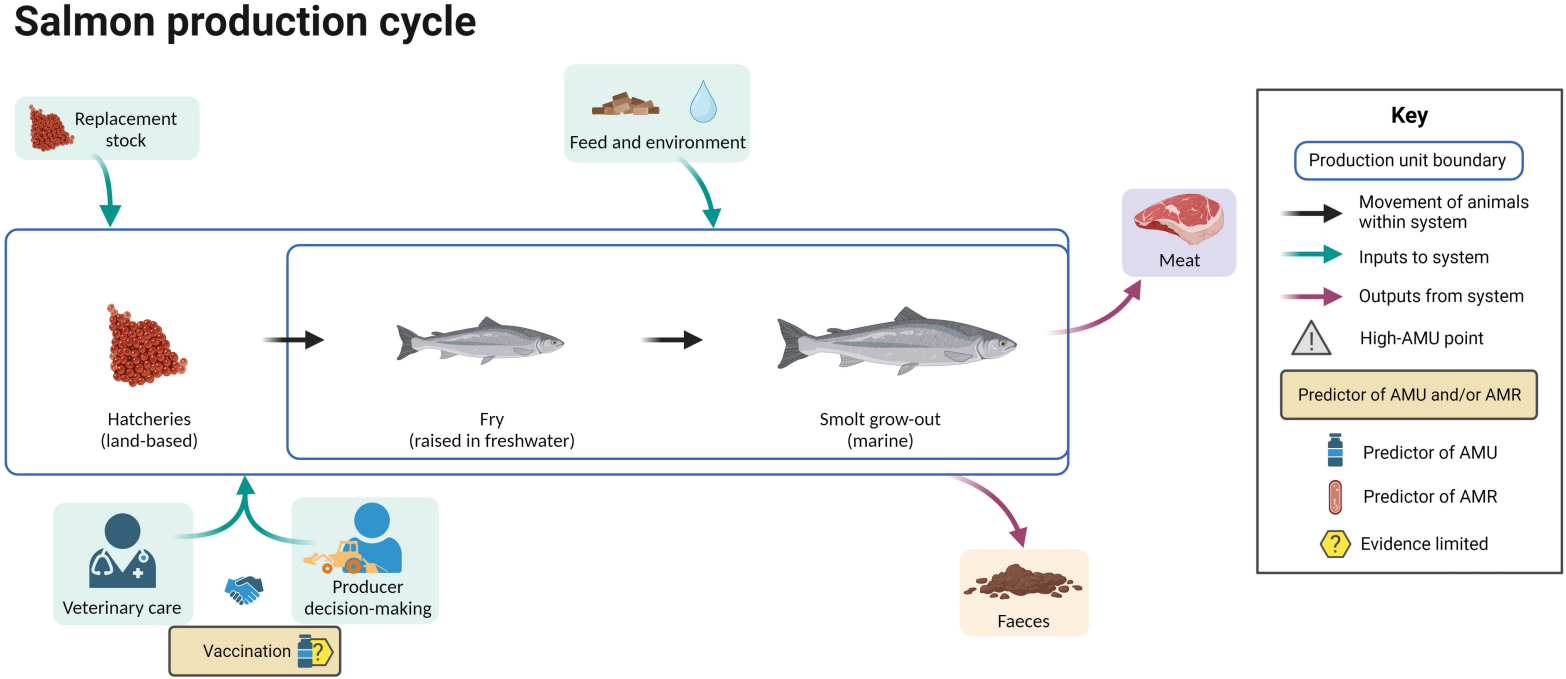
Factors associated with AMU in salmon aquaculture (no publications were identified addressing the predictors of AMR in this species). Created with BioRender.com.

**Figure 10** summarizes the available evidence regarding factors associated with AMU at different points in the salmon production cycle. No peer-reviewed publications were identified addressing risk factors for AMR in salmon.

## 4 Discussion

### 4.1 Distribution of the literature

The high number of papers addressing pig, dairy cattle and broiler production compared to other livestock types may reflect concerns surrounding these production types as possible sources of AMR. In particular, high biomass of farmed pigs and broiler chickens (Food and Agriculture Organisation (FAO), 2022a), both of which are often farmed intensively, may result in these livestock types contributing to a large proportion of livestock AMU. Meanwhile, AMU in dairy cattle can vary greatly due to differing dry cow management strategies and antibiotic-containing waste milk which may not enter the human food chain is still used on many farms to feed calves (Higham et al., 2018; Bernier Gosselin et al., 2022). However, aquaculture is a rapidly growing industry worldwide, prompting concerns about its contributions to AMU and AMR (Henriksson et al., 2018; Food and Agriculture Organisation (FAO), 2022b). Whilst a range of approaches to AMU/AMR mitigation in aquaculture have been identified (Henriksson et al., 2018), the specific determinants of AMU and AMR in salmon aquaculture require further investigation. Studies investigating beef cattle, sheep, laying chickens and turkeys were scarce even among European countries with the economic resources for research and coordinated EU programs such as the EFFORT consortium (EFFORT Consortium, 2014). Geographical distributions were particularly uneven for sheep, with quantitative studies identified only in the UK and Greece, and salmon, for which the only peer-reviewed literature was from Norway.

Even amongst the best-researched species there are significant gaps in our understanding of the impacts of key measures such as vaccination. The gaps seen in these high-income countries suggest that in low- and middle-income countries, literatures are likely to be even more limited, and livestock management and production system differences between global regions will likely limit the generalization from European results. The tendency for farms in Europe and other OECD countries to specialize in a specific species, and sometimes specific production stages, may not only make it difficult to generalize to other parts of the world but also encourage a more siloed approach in research.

### 4.2 Key findings and themes across production systems

Unsurprisingly, biosecurity measures were widely found to be associated with lower AMU and AMR. Introduction and spread of pathogens from new stock or *via* fomites might be expected to increase the need for therapeutic AMU, while AMR could be selected for by increased AMU as well as resistant organisms being introduced onto farms.

Organic status was generally associated with lower AMU, although the data available were extremely limited. Furthermore, continuing changes in farming practices and AMU regulations in both conventional and organic farms limit the conclusions that can be drawn from older studies on this topic. The association between organic status and AMR was clearer in some species than in others, and for sheep and beef cattle no studies were found to address this. In pigs and broiler chickens, organic production was more strongly associated with lower AMR than in other species. This may be in part due to the high AMU and intensive approaches often seen in conventional systems in these species, resulting in a greater difference in management between organic and conventional species. This possibility is supported by studies of farms in the USA and Canada, where conventional agriculture includes broader applications of antimicrobials, including for growth promotion, while animals must have zero AMU to be considered organic. In a USA study, for example, resistance to multiple antimicrobials was significantly more prevalent in turkeys and broiler chickens raised in conventional than organic systems (Luangtongkum et al., 2006). In dairy cattle, no clear AMR differences were detected between organic and conventional farms in studies in Europe or in New Zealand (McDougall et al., 2021), which has production systems more akin to Europe than North America. In contrast, significant AMR differences between organic and conventional dairy systems were observed in three North American studies (Tikofsky et al., 2003; Halbert et al., 2006; Rovira et al., 2019). These studies, and the repeated findings of AMR in animals not exposed to antimicrobials, highlight the complexity of the relationship between AMU and AMR in livestock.

Behavioral and social influences on AMU were frequently identified. “Treatment thresholds” varied between individual veterinarians and farmers, and in the few cases where it was investigated, reduced AMU and higher treatment thresholds did not appear to be associated with increased morbidity (Valle et al., 2007). This may be associated with situations in which non-antimicrobial treatment options such as supportive therapy may be more appropriate in the first instance. Antimicrobials are sometimes used inappropriately for prophylactic use due to concern that health, welfare and economic performance may otherwise be compromised (Doidge et al., 2021b). Prophylactic use of feed supplements and other alternatives, which were associated with lower AMU (Arnold et al., 2016; Adam et al., 2019), may meet the same need without overusing antimicrobials, although a recent survey of UK organic farmers suggested many were hesitant to try alternatives to antibiotics (Chylinski et al., 2022).

Economic concerns were frequently identified as a barrier to reducing AMU among farmers across different livestock types (Visschers et al., 2014; Doidge et al., 2020; Van Asseldonk et al., 2020; Doidge et al., 2021b). However, farm-specific interventions were found to be successful in reducing AMU without affecting economic performance (Rojo-Gimeno et al., 2016; Collineau et al., 2017b; Roskam et al., 2019) and an observational study found no association between prophylactic AMU in lambs and economic productivity (Lima et al., 2019). Estimates of economic costs and benefits of AMU across production systems have varied (OECD Working Party on Agricultural Policies and Markets, 2015; So et al., 2016) and require further research to address farmers’ financial concerns regarding AMU reduction.

Veterinarians’ prescribing behavior has been investigated to an extent, but some findings appeared discordant. Younger farm veterinarians in the UK showed a higher readiness to prescribe antibiotics for cattle and sheep without a farm visit (Doidge et al., 2019), while an international survey found that concern for AMR was negatively associated with number of years of experience in the cattle industry (Llanos-Soto et al., 2021). This could be a country effect, or younger veterinarians might feel more pressure and expectation to prescribe. Either way, this area merits further investigation as in Europe in particular, veterinarians are important gatekeepers of antimicrobials. Positive relationships between veterinarians and farmers tended to be associated with better farmer knowledge of AMR and multiple studies showed that bespoke herd health and AMU reduction plans developed in collaboration with farmers were successful in reducing AMU (Rojo-Gimeno et al., 2016; Collineau et al., 2017b; Roskam et al., 2019).

Several surprising findings emerged, most notably regarding the effects of vaccination on AMU in terrestrial livestock. The impacts of vaccination were in most cases small or non-significant, and in the case of infectious bursal disease in broilers, vaccination was positively associated with therapeutic AMU. In the latter case, a temporary immunosuppressive effect of vaccination has been reported, and this may transiently increase vulnerability to disease. In addition, vaccination may be more likely to be used on farms in which infectious disease is an existing problem.

Another counterintuitive finding was that in several species, internal biosecurity practices, particularly those relating to cleaning, were positively associated with AMU or AMR. Several possible explanations have been proposed: firstly, farms using additional cleaning protocols or isolation pens for sick animals may be doing so in response to an existing disease problem on-farm. Secondly, poorly implemented hygiene practices could increase bacterial spread, for example by using contaminated cleaning materials. Co-selection for resistance has also been identified as a possible mechanism by which disinfection with biocides may increase AMR.

### 4.3 Species-specific predictors

Free-range production appeared to have different effects in chickens compared to pigs. In chickens, the limited available data suggested that free-range layer systems were associated with higher AMU and unclear effects of free-range broiler production on AMU and AMR. In pigs, organic and conventional free-range systems tended to demonstrate significantly lower AMU and AMR than intensive farms, although there was little difference between the two types of free-range system. The reasons for the apparent differences in effect between livestock types merit further investigation.

Studies in dairy cattle consistently showed that feeding of antibiotic-containing waste milk was associated with at least a transient increase in AMR. Milk characteristics, including colostrum microflora, have been shown to impact calf gut microbiome and resistome, although progressive resistome changes have been shown to occur in correlation with gut maturation, with a tendency for AMR to decrease with age (Liu et al., 2019; Xue et al., 2021).

Finally, despite the sector overall reporting high AMU per unit biomass, a substantial number of broiler chicken flocks were raised without any use of antimicrobials, including in intensive systems. This may be possible due to short production cycles and high biosecurity, but even with low or zero AMU, the apparent dissemination of AMR bacteria through the broiler production chain highlights the importance of AMR control methods beyond simply removing the selection pressure.

### 4.4 Strengths and limitations

In this review, the links between AMU and AMR in livestock, and – to a lesser extent – between livestock AMU/AMR and AMR in humans, are assumed to be important. Certainly, if we are to address the challenges presented by AMR, a One Health approach is necessary to identify the epidemiological characteristics and potential drivers of AMR. However, it is important to recognize that correlates of AMU and AMR may not be determinants, and that many of the identified predictors may correlate with one another, resulting in confounding. In addition, the literature reviewed here highlights gaps and uncertainties in the links between AMU and AMR in livestock. Modelling suggests that curtailing livestock AMU alone would be unlikely to have a great effect on AMR in humans. The same study predicts that AMR transmission rates between humans and other animals, which are beyond the scope of this review, are likely to be an important determinant of the impact of any intervention designed to mitigate AMR in humans (Van Bunnik and Woolhouse, 2017).

A range of search term combinations were assessed when designing the search strategy for this review, of which the search used was the most suitable and included three major databases of scientific publications. Despite this, a high proportion of papers were found through reference list searching rather than the initial database search, suggesting that more literature may have been missed. Future reviews of this area are likely to benefit from a more refined search strategy. The livestock categories chosen for inclusion omitted species such as rabbits not typically farmed in the UK. Similarly, consultation with experts was UK-biased and due to this and the English-language criterion, grey literature included in the review was primarily British in origin. The differences between types of livestock and production systems may be marked between countries, and as a result the overrepresentation of UK literature could bias the findings of this review toward conditions and intervention outcomes in the UK. Whilst EU laws apply across the bloc, their implementation strategies have varied between countries and with the move of the UK away from the EU, policy differences are likely to grow between this and other nations.

However, this review employed a semi-systematic snowballing approach to investigate the peer-reviewed literature on this vital topic and to synthesize the existing knowledge on a wide range of predictors of livestock AMU and AMR in Europe. Interventions to mitigate AMU and AMR in livestock are likely to be relatively context-specific, but recurring themes identified in this review represent widely relevant areas of interest, such as biosecurity. The evidence included here may also facilitate more detailed country-level discussions of farming interventions, and allow comparison of findings between countries with more or less similar AMU and AMR policies and livestock production systems. Although they necessitate inclusion of a broader range of quality of material than would be included in a systematic review, the broad inclusion criteria allow the review to benefit from material that sheds light on areas in which little evidence is available. To the authors’ knowledge, this is the first comprehensive review of predictors and potential determinants on AMU and AMR in livestock, filling an important research gap and highlighting areas requiring further research.

## 5 Conclusion

This paper investigates and draws together the existing literature covering predictors of AMU and AMR at farm level in livestock in Europe. Key points in the production cycle and predictors for AMU and AMR identified for each livestock category are incorporated into visualizations of each production system as part of a systems approach to mitigating AMR. Even among the best-researched livestock types, pigs, broiler chickens and dairy cattle, substantial evidence gaps were identified. Future research should examine the impacts of vaccinations and other preventative health measures on AMU and AMR, investigate why some internal biosecurity practices seem to have negative effects and quantify the impacts of organic farming systems on AMR. In addition to addressing these gaps and expanding the research on beef cattle, laying hens, turkeys, sheep, and salmon, it is crucial that investigation of farm-level determinants of AMU and AMR should not be limited to high-income countries but include the low- and middle-income countries in which livestock production is most rapidly expanding.

## Author contributions

All four authors contributed to conception and design of the study. CR-W carried out the literature search and initial draft, including figures. DM, AP and AM supervised and provided feedback, making substantial contributions to manuscript revision. All authors contributed to the article and approved the submitted version.
